# SMURF2 attenuates NRF2-driven tumor progression by acting as a nuclear brake on NRF2 during cellular stress

**DOI:** 10.1016/j.redox.2026.104102

**Published:** 2026-02-28

**Authors:** Wanting Xu, Lei Dong, Jiaqian Li, Shuai Fan, Yadong Wang, Qin Xia

**Affiliations:** a Advanced Technology Research Institute, State Key Laboratory of Hearing and Balance Science and Key Laboratory of Molecular Medicine and Biological Diagnosis and Treatment (Ministry of Industry and Information Technology), Aerospace Center Hospital, Tangshan Research Institute, School of Life Science, Beijing Institute of Technology, Beijing, 100081, China; b Department of Infectious Diseases, The Hebei Medical University Third Hospital, Shijiazhuang, 050051, China

**Keywords:** SMURF2, NRF2, Protein aggregates, UPS, Nucleus ubiquitination, Glioblastoma

## Abstract

Constitutive activation of the transcription factor NRF2 confers therapeutic resistance in glioblastoma (GBM), however, this hyperactivation frequently persists despite the presence of intact KEAP1, suggesting the existence of KEAP1-independent regulatory mechanisms. Here, we identify the E3 ubiquitin ligase SMURF2 as a key nuclear regulator that restricts NRF2 activity and attenuates tumor progression. We demonstrate that SMURF2 overexpression suppresses the NRF2-mediated adaptive response to oxidative and proteotoxic stress, thereby reducing protein aggregation and promoting apoptosis. Mechanistically, cellular stress triggers the nuclear translocation of SMURF2, where it interacts with and degrades nuclear NRF2 via K48-linked polyubiquitination, independently of the canonical KEAP1 pathway. Consequently, a mutation in the nuclear localization sequence (NLS) of SMURF2 prevents its nuclear localization and fails to degrade NRF2. Additionally, the expression of an ubiquitination-resistant NRF2 mutant (K555R) prevents NRF2 degradation and abolishes stress-induced apoptosis. Clinically, high SMURF2 expression correlates with improved survival in patients with GBM exhibiting constitutive NRF2 activation. These findings uncover a novel axis of NRF2 regulation and highlight SMURF2 as a potential therapeutic target for NRF2-driven malignancies.

## Graphical abstract

Dysfunction of the KEAP1-dependent NRF2 degradation pathways results in nuclear accumulation of NRF2, driving cancer drug resistance and tumor progression. We identify SMURF2 as a nuclear “brake” that blocks NRF2-mediated stress adaptation and induces apoptosis. High SMURF2 levels correlate with better survival in glioblastoma patients with constitutive NRF2 activation, suggesting a tumor-suppressive role for SMURF2 in cancers addicted to NRF2 signaling.

**Figure:**
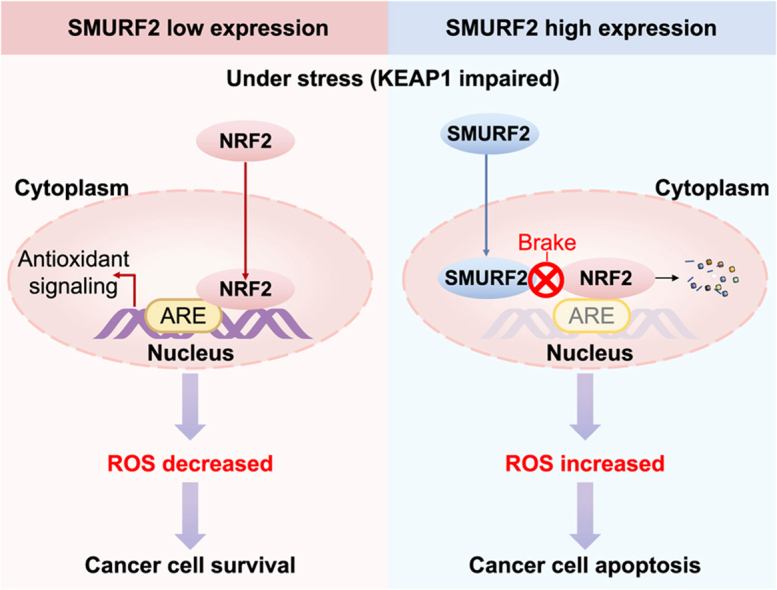

## 1 Introduction

The CNC-bZIP transcription factor NRF2 is the master regulator of the cellular defense against oxidative and proteotoxic stress [1]. Upon activation, NRF2 drives the expression of antioxidant response element (ARE)-dependent genes and maintains proteostasis by promoting p62/SQSTM1-mediated aggresome formation to sequester misfolded proteins [2]. To ensure homeostatic balance, NRF2 levels are tightly restricted by the ubiquitin-proteasome system, resulting in a half-life of less than 10 min under basal conditions [3]. This rapid turnover is primarily governed by two cytoplasmic E3 ligase complexes acting on distinct temporal scales. First, Kelch-like ECH-associated protein 1 (KEAP1) acts as a redox sensor to facilitate polyubiquitination of NRF2 for proteasomal degradation [4]. Under normal conditions, the KEAP1 homodimer binds NRF2 via the Neh2 domain (low-affinity with DLG motif and high-affinity with ETGE motif), facilitating its ubiquitination by the CUL3/RBX1 complex [5]. Second, the β-transducin repeat-containing protein (β-TrCP)/CUL1 axis provides a delayed feedback mechanism. Following the initial stress response, kinases such as GSK3 phosphorylate the NRF2 Neh6 domain, creating a phosphodegron recognized by β-TrCP, which drives nuclear export and degradation to terminate signaling by subsequent NRF2 polyubiquitination mediated by the CUL1 complex [6]. Together, the spatiotemporal dynamics of KEAP1 (acute cytoplasmic sensor) and β-TrCP (delayed terminator) ensure precise, self-limiting NRF2 activation [7].

Constitutive NRF2 activation is a hallmark of aggressive malignancies, driving therapeutic resistance and proliferation. For example, somatic mutations in *KEAP1* or *NFE2L2* drive this phenotype in lung cancer [8]. TCGA analysis reveals that Glioblastoma (GBM) lacks somatic *KEAP1/NFE2L2* mutations [9], yet patient specimens and cell lines exhibit persistent NRF2 hyperactivation [10]. Notably, pan-cancer evidence reveals that when PI3K signaling is hyperactivated, it synergistically coincides with elevated NRF2 activity, enabling tumor proliferation through PI3K-driven anabolic growth while NRF2 counters resultant oxidative damage [11]. This cooperativity is mechanistically reinforced by PI3K/AKT-mediated suppression of the β-TrCP degradation axis—where AKT-dependent GSK3β inhibition functionally impairs β-TrCP's ability to target NRF2 [12]. Furthermore, β-TrCP expression is markedly suppressed in GBM tissues, correlating with advanced tumor grade and poor survival [13]. With the collapse of these cytoplasmic degradation pathways, NRF2 accumulates in the nucleus [10]. This exposes a critical mechanistic gap: how do cancer cells evade nuclear termination of NRF2 signaling when cytoplasmic safeguards fail?

To address this, we investigated potential nuclear regulators capable of constraining pathological NRF2 activity. The HECT-domain E3 ubiquitin ligase SMURF2 is a potent tumor suppressor implicated in genomic stability, senescence, and cell cycle regulation [14]. SMURF2 colocalizes with ring finger protein 20 (RNF20) at γ-H2AX foci of double-stranded DNA breaks in the nucleus. It regulates histone modifications by targeting RNF20 for proteasomal degradation, thereby influencing histone H2B monoubiquitination and histone H3 trimethylation at Lys4 and Lys79. Importantly, a causal relationship between impaired nuclear function of SMURF2 and increased RNF20 expression has been observed in human tumors, suggesting a direct link between loss of SMURF2 function and cancer development [15]. Additionally, SMURF2 deficiency reduces p16 expression, impairs senescence response, and enhances susceptibility to spontaneous tumorigenesis, particularly B-cell lymphomas in mouse embryonic fibroblasts [16]. SMURF2 also negatively regulates several transcription factors implicated in carcinogenesis, drug resistance, and poor patient prognosis. For example, SMURF2 suppresses B-cell proliferation and lymphomagenesis by mediating ubiquitination and degradation of Yin Yang 1 (YY1), which is overexpressed in multiple cancer types [17]. Similarly, SMURF2 polyubiquitinates and promotes proteasomal degradation of Kruppel-like factor 5 (KLF5), inhibiting its transcriptional and pro-proliferative activities [18]. KLF5, involved in cell growth, survival, differentiation, migration, and stemness, exhibits abnormal expression in various cancers [19]. Given its capacity to target nuclear proteins for proteasomal degradation, we hypothesized that SMURF2 acts as a fail-safe mechanism to limit NRF2 stability. Here, we identify SMURF2 as a stress-inducible nuclear “brake” on NRF2. We demonstrate that SMURF2 translocates to the nucleus upon stress, where it directly binds and polyubiquitinates NRF2 at lysine 555 (K555), leading to KEAP1-independent degradation. We show that this axis suppresses NRF2-driven aggresome formation and promotes apoptosis, with high SMURF2 expression correlating with improved survival in GBM patients. These findings establish SMURF2 as a critical tumor suppressor that counters NRF2-mediated tumor progression.

## 2 Materials and methods

### 2.1 Cell culture, transfections, and drug treatments

The human glioblastoma cell lines LN229 and human embryonic kidney 293T (HEK293T) cell lines were purchased from the American Type Culture Collection (ATCC). The human embryonic kidney 293A (HEK293A) cell line was purchased from Invitrogen. Cells were cultured in Dulbecco's Modified Eagle's Medium (DMEM; Servicebio, G4511) supplemented with 10% fetal bovine serum (FBS; Hyclone, SH30406.05), and 1% penicillin-streptomycin (100 U/mL penicillin, 100 μg/mL streptomycin, Servicebio, G4003), at 5% CO_2_, 37 °C. Following the supplier's instructions, cells were transfected with plasmids using Lipofectamine 2000 (Invitrogen) and siRNA (JTSBIO Co., Ltd (Wuhan, China)) using lipofectamine RNAiMax (Invitrogen). The cells were collected and analyzed by western blot. Cells were treated with dimethyl sulfoxide (DMSO, Solarbio, D8371), Z-Leu-Leu-Leu-al (MG132, MCE, HY-13259), H_2_O_2_ (Beijing Tong Guang Fine Chemicals Company, 7722-84-1), Ivermectin (MCE, HY-15310), bafilomycin A1 (Baf-A1, Selleck, S1413) or Lipopolysaccharides (LPS; MCE, HY-D1056).

### 2.2 Antibodies

The primary antibodies used for western blot, immunofluorescence, and immunoprecipitation were as follows: anti-Flag (proteintech, 20543-1-AP), anti-HA (Abmart, M20013), anti-HA (Cell Signaling Technology, 3724), anti-His (proteintech, 66005-1-Ig), anti-GST (proteintech, 66001-2-Ig), anti-GFP (proteintech, 66002-1-Ig), anti-β-actin (Sigma-Aldrich, A1978), anti-α-Tubulin (proteintech, 66031-1-Ig), anti-Histone 2B (Santa CruZ, sc-515808), anti-Myc (proteintech, 16286-1-AP), anti-SMURF2 (Santa CruZ, sc-518164), anti-SMURF2 (Cell Signaling Technology, 12024), anti-NRF2 (proteintech, 16396-1-AP), anti-SQSTM1/p62 (MBL, PM045), anti-SQSTM1/p62 (Enzo life science, BML-PW9860), anti-cleaved-caspase-3 (Cell Signaling Technology, 9661), anti-Ubiquitin (Ub; MBL, D058-3), anti-Ubiquitin (Ub; ABclonal, A19686), anti-K48-linkage Specific Polyubiquitin (K48-Ub; Cell Signaling Technology, 4289).

The secondary antibodies for western blot were: HRP-conjugated Goat Anti-Rabbit IgG (H + L) (proteintech, SA00001-2), HRP-conjugated Goat Anti-Mouse IgG (H + L) (proteintech, SA00001-1). The secondary antibodies for immunofluorescence were: Alexa Fluor® 488 goat anti-rabbit IgG (Thermo Fisher Scientific, A11008), Alexa Fluor® 488 goat anti-mouse IgG (Thermo Fisher Scientific, A11001), Alexa Fluor® 555 goat anti-rabbit IgG (Thermo Fisher Scientific, A21428), Alexa Fluor® 555 goat anti-mouse IgG (Thermo Fisher Scientific, A21425), Coverslips were sealed with mounting medium containing 4′,6-diamidino-2-phenylindole (DAPI; Abcam, ab104139).

### 2.3 RNA interference and transfection

The siRNAs were purchased from JTSBIO Co., Ltd (Wuhan, China) and the following are the special sequences: SMURF2-siRNA (5′-CCUUCUGUGUUGAACAUAATT-3′), NRF2-siRNA (5′-GAAUGGUCCUAAAACACCATT-3′), KEAP1-siRNA (5′-TGGGGTCGTCGGTCTAGGG-3′). Cells were transfected with siRNA using lipofectamine RNAiMax (Invitrogen) according to the manufacturer's protocol. After 72 h of transfection, the cells were harvested, and the knockdown efficiency of the indicated proteins were detected by western blot assay.

The cells were transfected with the indicated plasmids for the overexpression experiment using Lipofectamine 2000 (Invitrogen) according to the manufacturer's protocol. After 24 h of transfection, the cells were lysed, and the efficiency of overexpression of the indicated protein was analyzed by a western blot assay.

### 2.4 Western blot

Protein expression levels were detected by western blotting. Cells were collected and lysed using RIPA buffer containing 1 mM PMSF (BOSTER, AR1192) and 1% phosphatase inhibitor (TargetMol, C0002), and the lysates were boiled with 5 × loading buffer. Proteins were separated by 10%-13.5% sodium dodecyl sulfate-polyacrylamide gel electrophoresis (SDS-PAGE) and electrophoretically transferred onto a nitrocellulose (NC) membrane. The membranes were then blocked with 5% nonfat milk at room temperature for 1 h, followed by incubation with primary antibodies at 4 °C overnight. After washing the NC membranes three times with 0.1% Tween-20/TBS (TBST), incubated with HRP-conjugated secondary antibodies at room temperature for 1 h. Following four washes with 0.1% TBST, the proteins were visualized using enhanced chemiluminescence (ECL) and quantified using ImageJ.

### 2.5 Immunofluorescence (IF)

The cells were seeded onto the circular microscope cover glasses in 12-well plates (1 × 10^5^ cells/well). Following treatment, cells were fixed with 4% paraformaldehyde for 30 min and subsequently permeabilized with 0.1% Triton X-100 for 5 min at room temperature. The cells were then blocked with 1% bovine serum albumin (BSA; Solarbio, A8020) for 1 h at room temperature and incubated with the primary antibody overnight at 4 °C. After washing with 0.1% Tween-20/PBS (PBST), the cells were incubated with fluorescent secondary antibody for 1 h at room temperature. DAPI solution was then added for 10 min of staining in the dark. Images were captured using a confocal laser scanning microscope (Nikon, N-SIM), and fluorescence intensity was quantified using ImageJ software.

### 2.6 Annexin V/FITC-PI staining

Cells were seeded in 12-well plates (1 × 10^5^ cells/well). After cell treatment, the cells were centrifuged at 1000×*g* for 5 min and discarded the supernatant. The cells were then resuspended in 1 mL pre-cooled PBS, centrifuged again at 1000×*g* for 5 min, and the discarded supernatant. Finally, the cells were resuspended in 195 μL Binding Buffer and added with 5 μL Annexin V-FITC and 10 μL PI Stain (Solarbio, CA1020). The cells were incubated in the dark for 10-20 min at room temperature. Apoptotic cells were detected using FACSAria™ III Cell Sorter (BD) flow cytometer and analyzed with FlowJo software.

### 2.7 Reactive oxygen species (ROS) assay

Cells were seeded in 12-well plates (1 × 10^5^ cells/well). After cell treatment, 10 μM DCFH-DA (Solarbio, CA1420) was incubated in serum-free DMEM to ensure complete coverage for 20 min at 37 °C. Subsequently, cells were washed three times with PBS. The cellular Relative levels of reactive oxygen species (ROS) were quantified using FACSAria™ III Cell Sorter and analyzed with FlowJo.

### 2.8 Nucleus and cytoplasm separation assay

The treatment cells were collected and lysed with Buffer A (pH 7.9 10 mM HEPES, 10 mM KCl, 1 mM EDTA, 1 mM EGTA, 1 mM DTT) containing 0.6% NP-40 for 30 min on ice. Cells were centrifuged at 600×*g*, 4 °C for 15 min, the supernatant was collected as the cytoplasmic fraction. The residual precipitate was washed with Buffer A and centrifuged at 600×*g*, 4 °C for 10 min 3 times. The final precipitate was resuspended in Buffer C (pH 7.9 20 mM HEPES, 400 mM KCl, 1 mM EDTA, 1 mM EGTA, 1 mM DTT), subjected to ultrasonication, and then centrifuged at 12000 rpm, 4 °C for 10 min to collect the nuclear extract in the supernatant. Buffer A and Buffer C were supplemented with 1 mM PMSF and 1% phosphatase inhibitor before use.

### 2.9 Soluble/insoluble protein fractionation

The treated cells were collected and lysed using a supernatant lysis buffer (150 mM NaCl; 50 mM Tris, pH 8.0; 10% glycerol; 2% Triton X-100; 1 mM EDTA) supplemented with 1 mM PMSF and 1% phosphatase inhibitor. Soluble and insoluble protein fractions were separated by centrifugation at 12,000 rpm for 10 min at 4 °C. The supernatant was collected as the soluble protein fraction, while the pellet constituted the insoluble protein fraction. The insoluble fraction was then resuspended in supernatant lysis buffer containing 10% SDS, 1 mM PMSF, and 1% phosphatase inhibitor, followed by sonication. Both soluble and insoluble protein fractions were analyzed using western blotting.

### 2.10 *In vitro* GST-pull down assay

The purified GST-tagged proteins (purified from *E. coli*) were incubated with Glutathione Sepharose 4B (Solarbio, P2020) beads at 4 °C overnight. After this incubation, the beads were washed 6 times with cold 1 × PBS, at 3000×*g* 2 min each time at 4 °C. Subsequently, the beads were incubated with the second protein (purified from *E. coli*) at 4 °C overnight. Following this second incubation, the beads were rewashed six times with cold 1 × PBS under the same conditions. Finally, the samples were eluted with 2 × loading buffer, boiled at 98 °C for 10 min, and then analyzed by western blotting.

### 2.11 Immunoprecipitation (IP)

For IP and co-IP experiments, treated cells were lysed in IP lysis buffer at 4 °C and centrifuged at 12,000 rpm for 15 min. For IP of endogenous SMURF2, anti-SMURF2 antibody (Cell Signaling Technology, 12024) or an equivalent amount of isotype IgG control antibody was first incubated with rProtein G (Solarbio, R8300) overnight at 4 °C on a tube rotator. An equal amount of centrifuged cell lysate was then added to the antibody-bead complexes and incubated overnight at 4 °C on a tube rotator. For co-IP, the centrifuged supernatant or purified protein (purified from *E. coli*) was incubated overnight at 4 °C on a tube rotator with specific antibody-conjugated beads (Anti-HA Affinity Gel, Beyotime, P2287; Anti-Flag Affinity Gel, Beyotime, P2271; Anti-GFP-Tag Agarose conjugated, Abmart, M20014; Glutathione Sepharose 4B, Solarbio, P2020). Following incubation, the beads were washed 6 times with cold 1 × PBS at 3000×*g* for 2 min each at 4 °C. Subsequently, the beads were incubated with the second protein source (supernatant or purified protein) overnight at 4 °C. After this second incubation, the beads were washed again 6 times with cold 1 × PBS under identical conditions (3000×*g*, 2 min, 4 °C). Finally, bound proteins were eluted using 2 × loading buffer, boiled at 98 °C for 10 min, and analyzed by western blotting.

### 2.12 *In vivo* and *in vitro* ubiquitylation assay

For the *in vivo* ubiquitination assay, HEK293T cells were transfected with combinations of plasmids encoding GFP-p62, HA-NRF2, Flag-NRF2, HA-tagged NRF2 truncations or point mutants, along with Flag-tagged ubiquitin mutants (Ub-K48, Ub-K63, Ub-K48R, Ub-K63R) or Flag-tagged SMURF2 variants (WT, CS, CS^C716A^) and Flag vector controls. Following transfection, cells were treated with 10 μM MG132 (a proteasome inhibitor; MCE, HY-13259) for 12 h. Cells were then lysed in IP lysis buffer at 4 °C, centrifuged at 12,000 rpm for 15 min, and the centrifuged supernatant were incubated directly with specific antibody-conjugated beads (anti-GFP, anti-HA, or anti-Flag) overnight at 4 °C on a tube rotator. Without washing, purified GST or GST-SMURF2 protein (expressed in and purified from *E. coli*) was added directly to the bead-lysate mixture and incubation continued overnight at 4 °C on a tube rotator. Subsequently, the beads were washed extensively 6 times with cold 1 × PBS at 3000×*g* for 2 min each at 4 °C. Bound proteins were eluted with 2 × SDS loading buffer, boiled at 98 °C for 10 min, separated by SDS-PAGE, and ubiquitination was analyzed by western blotting using anti-Flag or anti-Ub antibodies.

For the *in vitro* ubiquitination assay, His-Flag-NRF2 (WT or K555R) protein was incubated with 20 μL of anti-Flag beads overnight at 4 °C. The beads were then washed three times with cold 1 × PBS, followed by one wash with ubiquitin ligase buffer. Subsequently, the beads were incubated at 30 °C for 2 h in a ubiquitin ligase reaction mixture containing GST or GST-SMURF2, ATP, His-Ub, E1 enzyme, and E2 enzyme (UbcH5c). Finally, the proteins were added with 5 × SDS sample buffer and analyzed by western blotting.

### 2.13 Cycloheximide (CHX) chase assay

The cycloheximide (CHX) chase assay was recognized and used to detect intracellular protein degradation. Cells were transfected with HA-SMURF2 or SMURF2 siRNA plasmid in separate experiments, treated with CHX (100 μg/mL) for the indicated time (0, 4, 8, 12 h) and analyzed by western blotting.

### 2.14 Quantitative real-time PCR (qRT-PCR)

Cells were lysed in TRNzol Universal reagent (TIANGEN, DP424) and the total RNA was extracted according to manufacturer's protocol. 1 μg of total RNA was used as template for reverse transcription using Fast King RT Kit (With gDNase) (TIANGEN, KR116-02).10 ng of cDNA was used as template for qRT-PCR with SYBR green PCR mix (ABclonal, RK21203) and qRT-PCR was performed in Applied Biosystems 7500.

### 2.15 Primary cell culture

Primary cells were cultured from fresh patient-derived tissues. During transport, tissues were maintained on dry ice. Upon arrival, the tissues were minced into small fragments and washed three times with 1 × PBS containing antibiotics (100 U/mL penicillin and 100 μg/mL streptomycin). The fragments were then digested with EDTA-trypsin at 37 °C. After 10 min of digestion, the resulting cell suspension was collected, centrifuged, and the pellet was resuspended in fresh culture medium. Finally, the cells were placed in a humidified incubator at 37 °C with 5% CO_2_ for propagation.

### 2.16 Colony formation assay

Cells were seeded in 6-well plates (2000 cells/well). After 48 h, the cells were treated with or without LPS (100 ng/mL) for 12 h. Following treatment, the medium was replaced with fresh complete medium, and the cells were allowed to grow undisturbed for approximately 14 days to form colonies. The experiment was terminated when visible colonies had formed. Colonies were fixed with either 4% paraformaldehyde or methanol for 15 min at room temperature and then stained with 0.1% crystal violet for 30 min. Subsequently, the plates were gently washed with distilled water to remove excess stain, air-dried, and scanned for documentation and analysis.

### 2.17 Animal experiment

The animal experiments were conducted in accordance with a protocol approved by the Institutional Animal Care and Use Committee of Beijing Institute of Technology. Patient-derived GBM specimens were collected from surgical resections after obtaining informed consent and with approval from the relevant institutional ethics committee. Patient-derived cells (PDCs) transfected with either shPLKO (control) or shSMURF2 lentiviruses were suspended in a mixture of PBS and 20% Matrigel. Mice were injected subcutaneously with 1 × 10^7^ cells (in a 100 μL suspension) into both flanks. Upon tumor establishment, the mice were randomly allocated into two treatment groups (n = 5 per group). Beginning 14 days post-inoculation, mice received intraperitoneal injections of either PBS (vehicle control) or LPS (10 mg/kg) every two days. Tumor dimensions were measured daily using calipers, and tumor volume was calculated using the formula: Volume = 0.5 × L × W^2^ (where L is the longest diameter and W is the shortest diameter). Mice were euthanized at the experimental endpoint, after which tumors were excised, weighed, and processed for subsequent analysis.

### 2.18 Bioinformatics analysis

Gene expression profiles and clinical data regarding overall survival for the TCGA-GBMLGG cohort (n = 702) were obtained from https://xenabrowser.net/datapages/. A subset of samples (n = 186) exhibiting high NRF2 (*NFE2L2*) expression (above median) and low KEAP1 expression (below median) was identified. Within this “NRF2 high/KEAP1 low (NRF2^hi^/KEAP1^low^)” subset, samples were further stratified by SMURF2 expression into “High SMURF2” (n = 47, top 25%) and “Low SMURF2” (n = 139) groups. SMURF2 expression levels were visualized using boxplots, and statistical comparisons were performed using Student's t-test with *p* < 0.05 considered significant. Overall survival (OS) data were merged with gene expression profiles. Kaplan-Meier survival curves were generated, and univariate Cox proportional hazards regression was performed to assess the prognostic impact of SMURF2 expression levels.

For multivariable analysis, patients were filtered to include only high-grade gliomas (WHO grade III and IV). After merging clinical data and excluding cases with missing tumor grade information, 158 patients with complete clinical annotations were retained for analysis. High-grade gliomas (grade III/IV) were specifically selected for the forest plot analysis to minimize confounding effects of tumor grade heterogeneity. Multivariable Cox proportional hazards models were constructed adjusting for SMURF2 expression group (high vs. low), age categories (<40, 40-60, >60 years), gender (male vs. female), chemotherapy status (yes vs. no), and radiotherapy status (yes vs. no). Hazard ratios (HR) with 95% confidence intervals (CI) were calculated for each covariate, with p-values determined using the Wald test. The reference categories were: SMURF2-low, age <40 years, male gender, no chemotherapy, and no radiotherapy.

### 2.19 Statistical analysis

All results are presented as mean ± standard deviation (SD) from at least three replicates. The unpaired two-tailed Student's t-test was used to compare the two groups. The graphical presentations were created using Microsoft Office Excel. NS, not significant; ∗*p* < 0.05; ∗∗*p* < 0.01; ∗∗∗*p* < 0.001.

## 3 Results

### 3.1 SMURF2 prohibits the stress-mediated formation of ub^+^/p62^+^ aggresomes

**Fig. 1:**
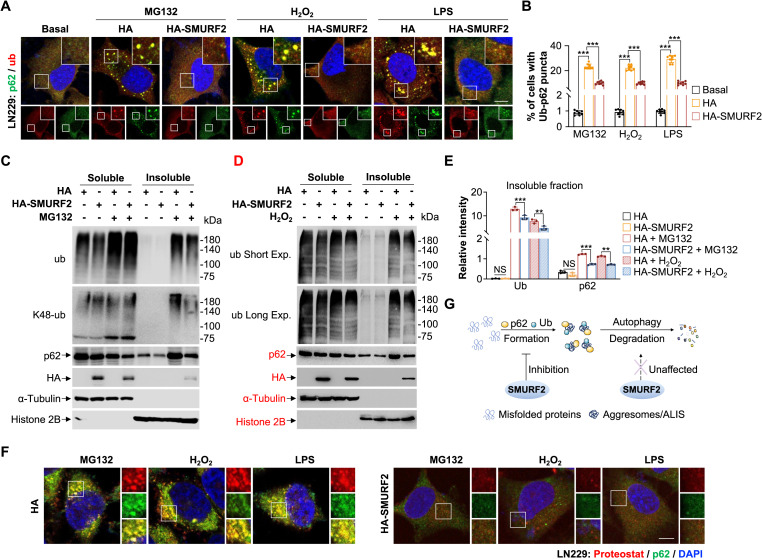
SMURF2 prohibits the stress-mediated formation of ub^+^/p62^+^ aggresomes. **(A and B)** LN229 cells were transfected with HA or HA-SMURF2 and treated with DMSO; MG132 (10 μM, 12 h); H_2_O_2_ (200 μM, 2 h); or LPS (100 ng/mL, 12 h). Representative immunofluorescence (IF) images of the colocalization of ub and p62. Nuclei stained with DAPI (A). Quantification the percentage of cells with ub^+^/p62^+^ puncta (B). **(C**–**E)** HEK293T cells were transfected with HA or HA-SMURF2 and subsequently treated with MG132 (10 μM, 12 h) or H_2_O_2_ (200 μM, 2 h). Detergent-soluble and detergent-insoluble fractions were analyzed by western blotting with indicated antibodies (C and D). Quantification of the relative intensity of ub and p62 levels in the detergent-insoluble fractions shown in C and D (E). **(F)** LN229 cells were transfected with HA or HA-SMURF2 and treated with DMSO; MG132 (10 μM, 12 h); H_2_O_2_ (200 μM, 2 h); or LPS (100 ng/mL, 12 h). Representative IF images of the colocalization of Proteostat and p62. Nuclei stained with DAPI. **(G)** The proposed model suggests that SMURF2 inhibits the formation of ub^+^/p62^+^ aggresomes/ALIS under stress conditions. Data were presented as the mean ± SD from three independent experiments. NS: not significant, ∗∗*p* < 0.01, ∗∗∗*p* < 0.001. Scale bar: 10 μm. Short Exp: short exposure; Long Exp: long exposure.

The consistency of oxidative or proteotoxic stress overwhelms the proteasome's ability to degrade misfolded proteins, cells sequester these proteins into aggresomes to preserve normal cellular function [20,21]. The autophagy adaptor SQSTM1/p62 facilitates this process by interacting with ubiquitinated misfolded proteins [22]. To investigate whether SMURF2 regulates the assembly or clearance of these aggregates, we monitored the colocalization of ubiquitin (ub) and p62 puncta. We found that SMURF2 overexpression significantly suppresses aggresome formation (ub^+^/p62^+^ puncta) under proteotoxic (MG132), oxidative (H_2_O_2_), and inflammatory (LPS) stress in LN229 cells (Fig. 1A and B). Consistently, biochemical fractionation revealed that HA-SMURF2 overexpression reduces the levels of ub and p62 in the insoluble fraction following stress treatment (Fig. 1C–E), suggesting SMURF2 overexpression either.

To distinguish whether SMURF2 inhibits the formation or enhances the autophagic degradation of aggregated proteins, we utilized exogenous, intrinsically aggregation-prone proteins. These included polyglutamine-expanded Huntingtin (Nhtt150Q and Nhtt60Q) [23], ALS-associated SOD1-G93A [24], and the TDP-43 fragment GFP-TDP25 [25]. Crucially, the aggregation of these proteins occurs independently of ubiquitination [[26], [27], [28]], making them ideal models to isolate degradation effects from formation interference. We found that neither HA-SMURF2 overexpression nor SMURF2 knockdown significantly altered the levels of these proteins in soluble or insoluble fractions (Fig. S1A–C), suggesting that SMURF2 does not promote the autophagic degradation of pre-formed aggregates. Consequently, we tested the hypothesis that SMURF2 prevents aggregate assembly using ProteoStat, a molecular rotor dye specific for aggregated protein structures. Immunofluorescence assays confirmed that HA-SMURF2 overexpression prevents the stress-induced colocalization of Proteostat with p62 in both LN229 and HEK293T cells (Fig. 1F and S1D). Collectively, these findings demonstrate that SMURF2 mitigates proteotoxic stress by impeding the formation of ub^+^/p62^+^ aggresomes (Fig. 1G).

Next, co-immunoprecipitation (Co-IP) assays demonstrated that SMURF2 indirectly interacts with p62 (Fig. S2A), and this interaction was significantly attenuated in response to H_2_O_2_ treatment (Fig. S2B). Furthermore, SMURF2 overexpression failed to mediate p62 ubiquitination, even in the presence of the proteasome inhibitor MG132 (Fig. S2C). These results suggest that SMURF2 suppresses aggregate formation via an alternative mechanism, rather than through direct interaction with or modification of p62 (Fig. S2D).

### 3.2 SMURF2 facilitates NRF2 proteasomal degradation in response to cellular stress

**Fig. 2:**
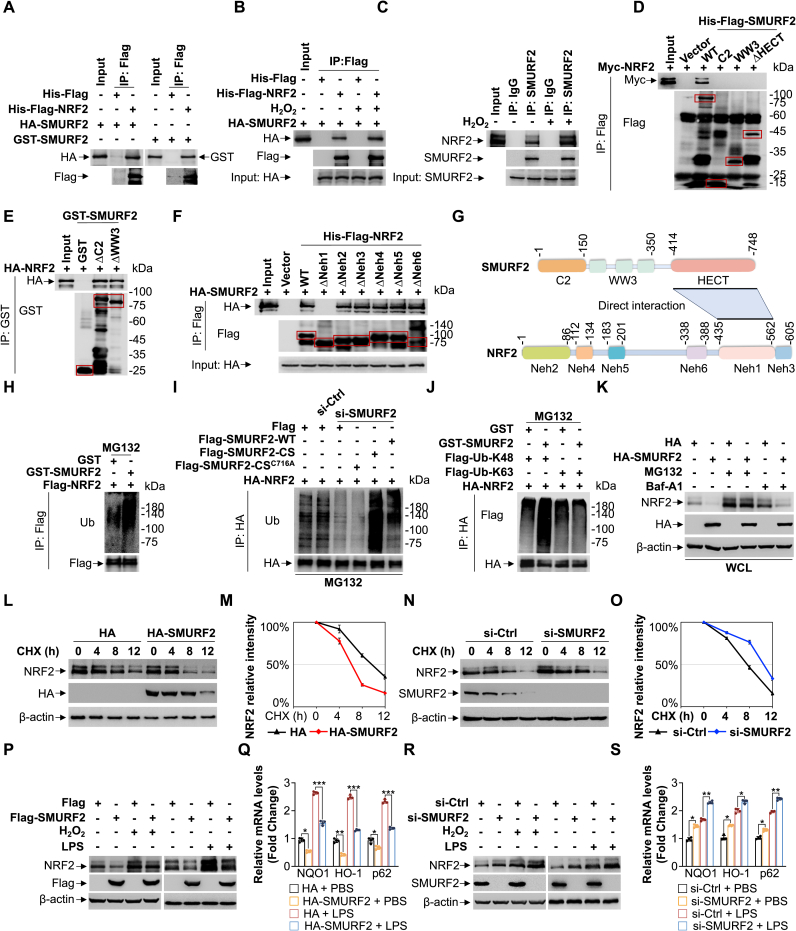
SMURF2 promotes NRF2 proteasomal degradation in response to cellular stress. **(A)** Co-immunoprecipitation (Co-IP) assay was performed to analyze the interaction between SMURF2 and NRF2. **(B)** Co-IP assay analysis of the interaction between HA-SMURF2 and His-Flag-NRF2 after treated with or without H_2_O_2_ (200 μM, 2 h) **(C)** Endogenous co-IP assay analysis of the interaction between endogenous SMURF2 and NRF2 in HEK293T cells after treated with or without H_2_O_2_ (200 μM, 2 h). **(D and E)** Co-IP assay analysis of the interaction between His-Flag-SMURF2 constructs (WT, C2, WW3 and ΔHECT) and Myc-NRF2 (D); the interaction between GST-SMURF2 constructs (ΔC2 and ΔWW3) and HA-NRF2 (E). **(F)** Co-IP assay analysis of the interaction between His-Flag-NRF2 constructs (WT and ΔNeh1-6) and HA-SMURF2. **(G)** Schematic diagram of mapping the direct interaction between SMURF2 and NRF2. **(H)** HEK293T cells expressing Flag-NRF2 were treated with MG132 (10 μM,12 h). The ubiquitination of Flag-NRF2 in the presence of purified GST or GST-SMURF2 was then detected by western blotting. **(I)** HEK293T cells were transfected with SMURF2 siRNA or scramble siRNA for 48 h, then transfected with HA-NRF2 and restored with Flag-SMURF2-WT/CS/CS^C716A^, treated with MG132 (10 μM, 12 h). Ubiquitination of HA-NRF2 was assessed by co-IP after SMURF2 knockdown and functional restoration. **(J)** HEK293T cells expressing HA-NRF2 and Flag-Ub-K48 or Flag-Ub-K63. The K48-linked or K63-linked ubiquitination of HA-NRF2 in the presence of purified GST or GST-SMURF2 was then detected by western blotting. **(K)** HEK293T cells were transfected with either HA or HA-SMURF2, then treated with DMSO, MG132 (10 μM, 12 h) or Bafilomycin A1 (Baf-A1, 100 nM, 6 h) and analyzed by western blotting of whole cell lysates (WCL) using the indicated antibodies. **(L**–**O)** HEK293T cells were transfected either with HA or HA-SMURF2 (L), or with SMURF2 siRNA or scramble siRNA oligos for 48 h (N), then treated with cycloheximide (CHX, 100 μg/mL) for the indicated times and analyzed by western blotting using the indicated antibodies. Quantification of the relative intensity of NRF2 is shown (M, O). **(P)** HEK293T cells were transfected with either Flag or Flag-SMURF2, then treated with PBS, H_2_O_2_ (200 μM, 2 h), or LPS (100 ng/mL, 12 h) and analyzed by western blotting using the indicated antibodies. **(Q)** HEK293T cells were transfected with either HA or HA-SMURF2, then treated with PBS or LPS (100 ng/mL, 12 h) and analyzed by qRT-PCR using primers specific for indicated genes. The fold change in expression in HA-SMURF2 overexpressing samples was calculated relative to control samples. **(R)** HEK293T cells were transfected with SMURF2 siRNA or scramble siRNA oligos for 48 h, then treated with PBS, H_2_O_2_ (200 μM, 2 h), or LPS (100 ng/mL, 12 h) and analyzed by western blotting using the indicated antibodies. **(S)** HEK293T cells were transfected with SMURF2 siRNA or scramble siRNA oligos for 48 h, then treated with PBS or LPS (100 ng/mL, 12 h) and analyzed by qRT-PCR using primers specific for indicated genes. The fold change in expression in si-SMURF2 samples was calculated relative to control samples. Data were presented as the mean ± SD from three independent experiments. ∗*p* < 0.05, ∗∗*p* < 0.01, ∗∗∗*p* < 0.001.

A well-established NRF2-p62 positive feedback loop has been described previously, in which NRF2 activates p62 transcription, while p62 stabilizes NRF2 by sequestering KEAP1 to prevent its ubiquitination [29,30]. This mechanism drives proteotoxic stress responses by promoting aggresome formation [2]. Co-IP and pull-down assays revealed a direct interaction between SMURF2 and NRF2 (Fig. 2A), which significantly increased under H_2_O_2_-induced oxidative stress (Fig. 2B). Importantly, endogenous NRF2 also exhibited a strong interaction with SMURF2 following H_2_O_2_ treatment (Fig. 2C). Domain mapping demonstrated that the HECT domain of SMURF2 and the Neh1 domain of NRF2 are critical for this direct interaction (Fig. 2D-G).

Given that SMURF2 is an E3 ubiquitin ligase [31], we assessed whether it targets NRF2 for ubiquitination. Indeed, we found that SMURF2 significantly promotes the ubiquitination of NRF2 (Fig. 2H). NRF2 ubiquitination could be rescued by restoration with Flag-SMURF2-CS (codon substitution; si-SMURF2 resistant), but not with Flag-SMURF2-CS^C716A^ (lacks E3 ubiquitin ligase activity [32]), in SMURF2-knockdown cells (Fig. 2I). These findings collectively demonstrate that SMURF2 directly interacts with NRF2 to promote its ubiquitination in an E3 ligase activity-dependent manner. Typically, K48-linked ubiquitination leads to the degradation of target proteins through the ubiquitin-proteasome system (UPS), while K63-linked ubiquitination directs target proteins toward degradation via autophagy [33]. To further investigate the form of polyubiquitin chains of NRF2 mediated by SMURF2, we employed two ubiquitin mutants: Flag-Ub-K48 and Flag-Ub-K63, which exclusively allow the formation of polyubiquitin chains at lysine-48 and lysine-63 residues, respectively. We found that SMURF2-mediated ubiquitination of NRF2 was enhanced in cells transfected with Flag-Ub-K48, but no significant change was observed with Flag-Ub-K63 (Fig. 2J). Additionally, transfection of the Flag-Ub-K48R mutant (prevents ubiquitination at lysine-48), but not the Flag-Ub-K63R mutant (blocks ubiquitination at lysine-63), suppressed SMURF2-mediated NRF2 ubiquitination (Fig. S3A and S3B). These results suggest that SMURF2 predominantly links K48 polyubiquitin chains to NRF2. Next, our results indicate that SMURF2 overexpression reduced NRF2 levels in whole-cell lysates (WCL) by using proteasome inhibitor MG132, but not by the autophagy inhibitor Bafilomycin A1 (Baf-A1) (Fig. 2K). These data collectively suggest that SMURF2 promotes NRF2 degradation specifically through K48-linked ubiquitination for proteasomal degradation. Indeed, cycloheximide (CHX) chase assays revealed that SMURF2 overexpression promoted NRF2 degradation in a time-dependent manner (Fig. 2L, quantified in Fig. 2M). Conversely, SMURF2 knockdown enhanced NRF2 stability (Fig. 2N, quantified in Fig. 2O), further confirming SMURF2's role in promoting NRF2 degradation. Previous data demonstrated that H_2_O_2_ enhances the interaction between SMURF2 and NRF2 (Fig. 2B). This finding prompted us to investigate whether SMURF2 degrades NRF2 under stress conditions. Subsequent studies revealed that Flag-SMURF2 overexpression led to a significant decrease in NRF2 levels upon H_2_O_2_ or LPS treatment (Fig. 2P). A hallmark of the NRF2-mediated cellular stress response is the upregulation of target genes such as NAD(P)H quinone dehydrogenase 1 (NQO1), heme oxygenase-1 (HO-1), and p62. Indeed, Flag-SMURF2 overexpression significantly reduced mRNA levels of NQO1, HO-1, and p62 under LPS stimulation (Fig. 2Q). Conversely, SMURF2 knockdown significantly increased NRF2 protein levels and the mRNA expression of its downstream target genes compared to controls under stress conditions (H_2_O_2_ or LPS) (Fig. 2R and S). Collectively, these data reveal that SMURF2 promotes NRF2 proteasomal degradation in response to cellular stress.

### 3.3 SMURF2 nuclear translocation facilitates the degradation of NRF2 within the nucleus

**Fig. 3:**
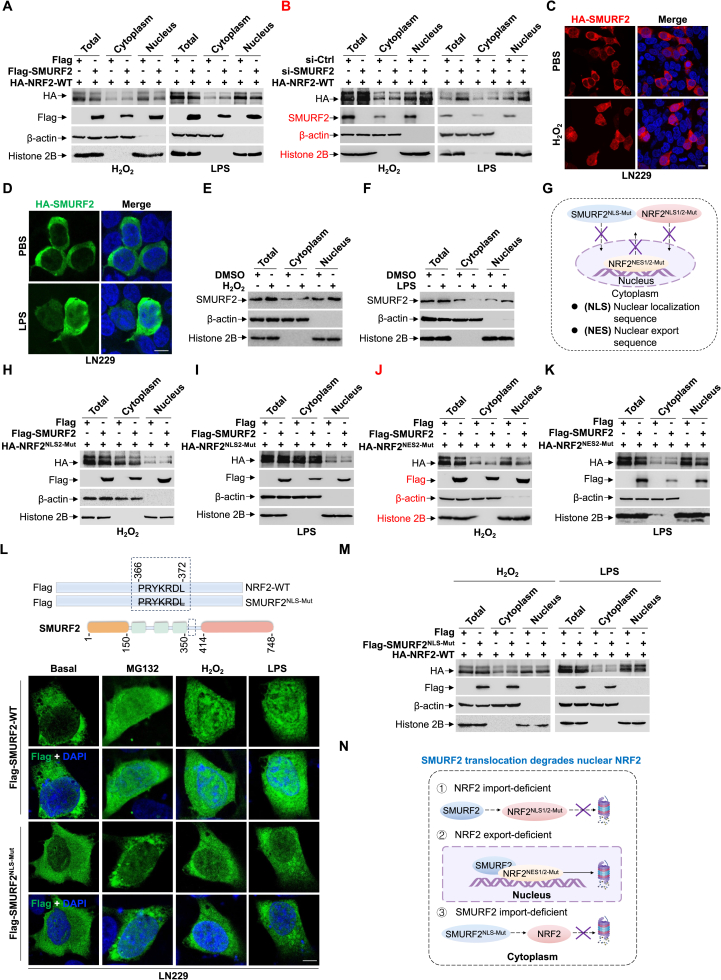
SMURF2 nuclear translocation facilitates the degradation of NRF2 within the nucleus. **(A and B)** HEK293T cells were first transfected with HA-NRF2-WT. Subsequently, cells were transfected either with Flag or Flag-SMURF2 (A), or with SMURF2 siRNA or scrambled siRNA oligos for 48 h (B). Cells were then treated with H_2_O_2_ (200 μM, 2 h) or LPS (100 ng/mL, 12 h). Following treatment, subcellular fractionation was performed to isolate total, nuclear, and cytoplasmic proteins, followed by western blot analysis with indicated antibodies. **(C and D)** LN229 cells were transfected with HA-SMURF2, then treated with PBS, H_2_O_2_ (200 μM, 2 h) (C) or LPS (100 ng/mL, 12 h) (D). Representative IF images showing the subcellular localization of HA-SMURF2 are presented; nuclei were stained with DAPI. **(E and F)** HEK293T cells were treated with PBS, H_2_O_2_ (200 μM, 2 h) (E), or LPS (100 ng/mL, 12 h) (F). Subcellular fractionation was performed to isolate total, nuclear, and cytoplasmic proteins, followed by western blot analysis with indicated antibodies. **(G)** Schematic depiction of NRF2^NLS1/2−Mut^, NRF2^NES1/2−Mut^, and SMURF2^NLS−Mut^. **(H–K)** HEK293T cells were first transfected with HA-NRF2^NLS2−Mut^ (H and I) or HA-NRF2^NES2−Mut^ (J and K). Subsequently, cells were transfected either with Flag or Flag-SMURF2, then treated with H_2_O_2_ (200 μM, 2 h) (H and J) or LPS (100 ng/mL, 12 h) (I and K). Following treatment, subcellular fractionation was performed to isolate total, nuclear, and cytoplasmic proteins, followed by western blot analysis with indicated antibodies. **(L)** Schematic of SMURF2 domains with NLS indicated (single-letter code). SMURF2^NLS−Mut^ denotes deletion of NLS residues (Top). LN229 cells transfected with Flag-SMURF2-WT or Flag-SMURF2^NLS−Mut^ were treated with DMSO, MG132 (10 μM, 12 h), H_2_O_2_ (200 μM, 2 h), or LPS (100 ng/mL, 12 h). Representative IF images showing subcellular localization of Flag-SMURF2 or Flag-SMURF2^NLS−Mut^; nuclei were stained with DAPI (Bottom). **(M)** HEK293T cells were first transfected with HA-NRF2-WT. Subsequently, cells were transfected either with Flag or Flag-SMURF2^NLS−Mut^, then treated with H_2_O_2_ (200 μM, 2 h) or LPS (100 ng/mL, 12 h). Following treatment, subcellular fractionation was performed to isolate total, nuclear, and cytoplasmic proteins, followed by western blot analysis with indicated antibodies. **(N)** The proposed model indicates that SMURF2 specifically degrades NRF2 in the nucleus. Scale bar: 5 μm, Scale bar: 10 μm. Data were presented in three independent experiments.

Given that the transcription factor NRF2 rapidly translocate to the nucleus under stress conditions, we wondered whether SMURF2 regulates NRF2 in the nuclear compartment. Nuclear-cytoplasmic fractionation assays revealed that overexpression of Flag-SMURF2 specifically promotes the degradation of nuclear NRF2 (Fig. S4A). Consistently, overexpression of Flag-SMURF2 significantly reduced nuclear, but not cytoplasmic, HA-NRF2 upon stress (Fig. 3A). Conversely, SMURF2 knockdown promoted the accumulation of nuclear NRF2 under stress conditions (Fig. 3B). We next examined the cellular localization of SMURF2 under stress conditions. IF and western blot assays demonstrated that SMURF2 is colocalized in both the cytoplasm and nucleus under basal conditions. Furthermore, SMURF2 exhibits enhanced translocation to the nucleus upon treatment with H_2_O_2_ and LPS (Fig. 3C-F). To further investigate whether SMURF2 interacts with NRF2 in the nucleus, we use the nuclear import inhibitor ivermectin, which binds to importin α, to disrupt importin α/β1-mediated nuclear import by interfering with the recognition of nuclear localization sequence (NLS) [34]. Indeed, ivermectin prevents the nuclear translocation of NRF2 following upon H_2_O_2_ and LPS treatment (Fig. S4B and S4C). Strikingly, we found that blocking nuclear translocation of Flag-SMURF2 in the presence of ivermectin fails to induce degradation of HA-NRF2 with or without H_2_O_2_ and LPS treatment (Fig. S4D–S4F).

The subcellular localization of NRF2 is controlled by its NLS and nuclear export sequence (NES) [35,36]. To further verify whether SMURF2-mediated degradation of NRF2 in the nucleus, we constructed a series of HA-tagged NRF2 localization mutants (Fig. 3G): HA-NRF2^NLS1−Mut^ (mutating the previously identified primary functional NLS, the RKRK motif at residues 515-518), HA-NRF2^NLS2−Mut^ mutants (mutating a putative secondary NLS region), HA-NRF2^NES1−Mut^ and HA-NRF2^NES2−Mut^ (mutating two distinct functional NES motifs) [36]. As reported previously study [35,37], HA-NRF2^NLS1−Mut^ exhibited both cytosolic and nuclear localization, whereas HA- NRF2^NLS2−Mut^ was exclusively cytosolic. Conversely, both HA-NRF2^NES1−Mut^ and HA-NRF2^NES2−Mut^ were forced to accumulate primarily in the nucleus due to mutations disrupting their respective NES (Fig. S4G and S4H). Consistently, western blot analysis revealed that overexpression of Flag-SMURF2 promoted HA-NRF2-WT, HA-NRF2^NES1−Mut^, and HA-NRF2^NES2−Mut^ degradation, but not HA-NRF2^NLS1−Mut^ and HA-NRF2^NLS2−Mut^ (Fig. S4I and S4J). Next, nuclear and cytoplasmic fractionation assay identifies overexpression of Flag-SMURF2 specifically degrades nuclear fraction of HA-NRF2^NES2−Mut^, but not HA-NRF2^NLS2−Mut^, upon H_2_O_2_ and LPS treatment (Fig. 3H-K), suggesting SMURF2 NLS facilitates its nuclear translocation. We then predicted the putative NLS in SMURF2 to be the 366-372 region by using the PSORT program [38] and constructed Flag-SMURF2^NLS−Mut^ (Flag-SMURF2-Δ366-372), a nuclear localization-deficient form of Flag-SMURF2 (Fig. 3G and L). IF assay verified that Flag-SMURF2^NLS−Mut^ loss its ability of nuclear translocation upon MG132, H_2_O_2_, and LPS treatment (Fig. 3L). Western blot analysis further demonstrated that overexpression of Flag-SMURF2^NLS−Mut^ did not result in the degradation of NRF2 in the nucleus under stress conditions, indicating that nuclear localization of SMURF2 is required for NRF2 degradation (Fig. 3M). Taken together, these results indicate that the nuclear translocation of SMURF2 is required for the degradation of nuclear NRF2 (Fig. 3N).

### 3.4 SMURF2 ubiquitinates NRF2 at K555 in the nucleus for its degradation

**Fig. 4:**
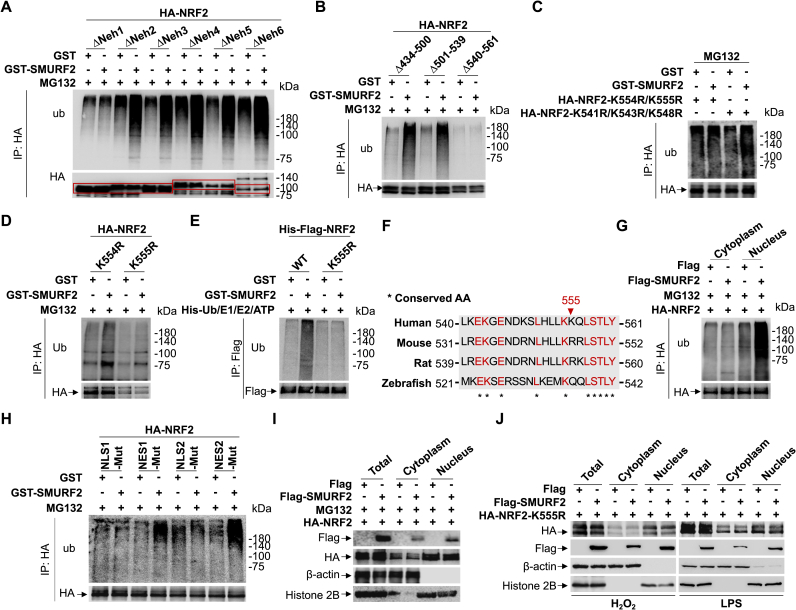
SMURF2 ubiquitinates NRF2 at K555 in the nucleus for its degradation. **(A)** HEK293T cells were transfected with HA-NRF2 constructs (ΔNeh1-ΔNeh6), followed by treatment with MG132 (10 μM, 12 h). The ubiquitination of HA-NRF2 constructs (ΔNeh1-ΔNeh6) in the presence of purified GST or GST-SMURF2 was then detected by western blotting. **(B)** HEK293T cells were transfected with HA-NRF2 constructs (Δ434-500, Δ501-539 and Δ540-561), followed by treatment with MG132 (10 μM, 12 h). The ubiquitination of HA-NRF2 constructs (Δ434-500, Δ501-539 and Δ540-561) in the presence of purified GST or GST-SMURF2 was then detected by western blotting. **(C)** HEK293T cells were transfected with HA-NRF2 constructs (K554R/K555R and K541R/K543R/K548R), followed by treatment with MG132 (10 μM, 12 h). The ubiquitination of HA-NRF2 constructs (K554R/K555R and K541R/K543R/K548R) in the presence of purified GST or GST-SMURF2 was then detected by western blotting. **(D)** HEK293T cells were transfected with HA-NRF2 constructs (K554R and K555R), followed by treatment with MG132 (10 μM, 12 h). The ubiquitination of HA-NRF2 constructs (K554R and K555R) in the presence of purified GST or GST-SMURF2 was then detected by western blotting. **(E)** Purified SMURF2 with His-ub, E1, E2 (UbcH5c), and ATP were used as indicated and evaluated by pull-down assay. Ubiquitinated NRF2-WT and K555R detected by immunoblotting against anti-Ub and anti-Flag. **(F)** Sequence alignment of NRF2 sites on CNA orthologs of different species. **(G)** HEK293T cells overexpressing HA-NRF2 were transfected with Flag or Flag-SMURF2, followed by treatment with MG132 (10 μM, 12 h). Following treatment, subcellular fractionation was performed to isolate cytoplasmic and nuclear fractions. The ubiquitination of cytoplasmic and nuclear fractions was then detected by western blotting. **(H)** HEK293T cells were transfected with HA-NRF2^NLS1−Mut^, HA-NRF2^NES1−Mut^, HA-NRF2^NLS2−Mut^ or HA-NRF2^NES2−Mut^, followed by treatment with MG132 (10 μM, 12 h). The ubiquitination of HA-NRF2 constructs in the presence of purified GST or GST-SMURF2 was then detected by western blotting. **(I and J)** HEK293T cells were first transfected with HA-NRF2 (I), or HA-NRF2-K555R (J), then were transfected with Flag or Flag-SMURF2, followed by treatment with MG132 (10 μM, 12 h) (I), H_2_O_2_ (200 μM, 2 h) or LPS (100 ng/mL, 12 h) (J). Following treatment, subcellular fractionation was performed to isolate total, nuclear, and cytoplasmic proteins, followed by western blot analysis with indicated antibodies. Data were presented in three independent experiments.

To further elucidate the mechanism underlying this regulation, we sought to identify the specific ubiquitination sites on NRF2 that can be modified by SMURF2. Our results demonstrated that SMURF2-mediated ubiquitination of NRF2 within Neh1 domain, evidenced by the presence of the ΔNeh1, but not ΔNeh2-6, prohibits SMURF2-mediated ubiquitination (Fig. 4A). Further analysis of the revealed that deletion of amino acids 540-561 (Δ540-561), but not 434-500 (Δ434-500) or 501-539 (Δ501-539) within Neh1 domain inhibited SMURF2-mediated NRF2 ubiquitination (Fig. 4B). To identify specific ubiquitination sites on NRF2 modified by SMURF2, we generated point mutants of NRF2 by individually replacing lysine (K) residues with arginine (R) within the 540-561 region of NRF2. Notably, only the K554R/K555R double mutation prevented SMURF2-mediated ubiquitination of NRF2 (Fig. 4C). To distinguish the contributions of these two sites, we generated the single-point mutants K554R and K555R and found that only the K555R mutation blocked SMURF2-mediated ubiquitination (Fig. 4D), and this effect was confirmed by an *in vitro* assay showing the mutant could no longer be ubiquitinated by SMURF2 (Fig. 4E). Interestingly, sequence alignment revealed that K555 is unique to human NRF2, suggesting a potentially human-specific mechanism for SMURF2-mediated regulation of NRF2 (Fig. 4F).

However, the precise regulation of subcellular location where NRF2 ubiquitination occurs remained unclear. To our knowledge, previous studies have not demonstrated nuclear ubiquitin-mediated degradation of NRF2. Notably, our subcellular fractionation experiments unexpectedly discovered that SMURF2 specifically ubiquitinates NRF2 in the nuclear compartment (Fig. 4G). Further analysis revealed that SMURF2 selectively ubiquitinates nuclear NRF2 mutants (HA-NRF2^NES1−Mut^ and HA-NRF2^NES2−Mut^) but not cytoplasmic mutants (HA-NRF2^NLS1−Mut^ and HA-NRF2^NLS2−Mut^), supporting the notion that SMURF2 promotes NRF2 ubiquitination in the nucleus (Fig. 4H). Moreover, Flag-SMURF2 overexpression failed to induce NRF2 degradation in the nucleus under treatment with MG132, indicating that SMURF2-mediated NRF2 degradation is proteasome-dependent (Fig. 4I). Importantly, Flag-SMURF2 overexpression failed to degrade HA-NRF2-K555R in the nucleus under stress conditions (Fig. 4J). Consistent with our previous findings, these results demonstrate that SMURF2 specifically promotes the ubiquitination and degradation of NRF2 at K555 in the nuclear compartment.

### 3.5 SMURF2 promotes cell apoptosis through NRF2 inactivation

**Fig. 5:**
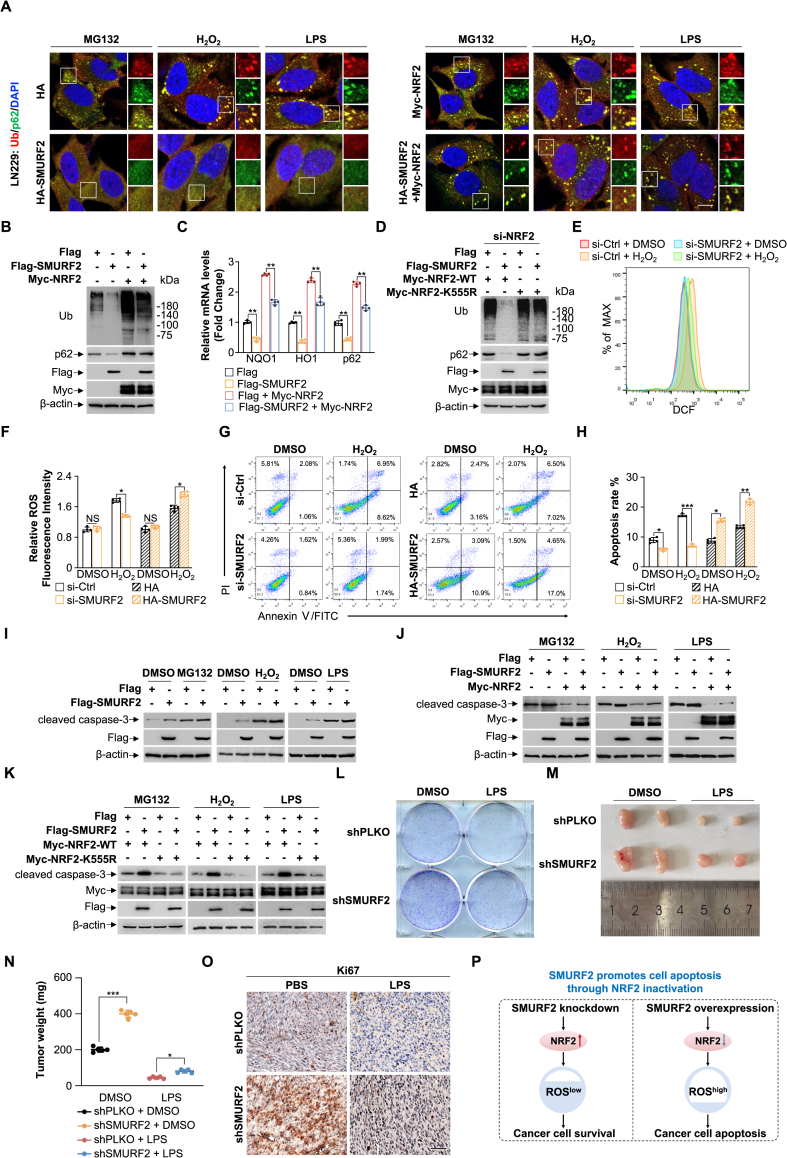
SMURF2 promotes cell apoptosis through NRF2 inactivation. **(A)** LN229 cells were transfected with HA, HA-SMURF2, Myc-NRF2 or HA-SMURF2 + Myc-NRF2 and subsequent treatment with MG132 (10 μM, 12 h), H_2_O_2_ (200 μM, 2 h), or LPS (100 ng/mL, 12 h). Representative IF images of the colocalization of ub and p62. Nuclei stained with DAPI. **(B and C)** HEK293T cells were transfected with empty vector or Myc-NRF2, followed by Flag or Flag-SMURF2 expression. Western blotting was performed using indicated antibodies (B). qRT-PCR was performed using primers specific for indicated genes (C). The fold change in expression in Flag-SMURF2 overexpressing samples was calculated relative to control samples. **(D)** HEK293T cells were first transfected with NRF2 siRNA for 48 h, followed by transfection with either Myc-NRF2-WT or Myc-NRF2-K555R, and subsequently transfected with Flag or Flag-SMURF2.Western blotting was performed using indicated antibodies. **(E)** LN229 cells were transfected with SMURF2 siRNA or scramble siRNA oligos for 48 h, followed by treatment with PBS or H_2_O_2_ (200 μM, 2 h). Cells were then stained with 2,7-Dichlorodihydrofluorescein diacetate (DCFH-DA, 10 μM) and the ROS level was detected by flow cytometry. **(F)** Quantification of relative ROS fluorescence intensity in LN229 cells with SMURF2 knockdown or HA-SMURF2 overexpression. **(G and H)** LN229 cells were transfected with SMURF2 siRNA or scramble siRNA oligos for 48 h or transfected with HA or HA-SMURF2, followed by treatment with PBS or H_2_O_2_ (200 μM, 2 h). Cells were then stained with Annexin-V/propidium iodide (PI), and apoptotic cells were detected by flow cytometry (G). Quantification of apoptosis in LN229 cells with SMURF2 knockdown or HA-SMURF2 overexpression (H). **(I)** HEK293T cells were transfection with Flag or Flag-SMURF2, and subsequent treatment with or without MG132 (10 μM, 12 h), H_2_O_2_ (200 μM, 2 h), or LPS (100 ng/ml, 12 h). Western blotting was performed using indicated antibodies. **(J and K)** HEK293T cells were transfected with empty vector and Myc-NRF2 (J) or Myc-NRF2-WT, Myc-NRF2-K555R (K), followed by Flag or Flag-SMURF2 expression. Cells were subsequently treated with MG132 (10 μM, 12 h), H_2_O_2_ (200 μM, 2 h), or LPS (100 ng/mL, 12 h). Western blotting was performed using indicated antibodies. **(L)** The shSMURF2 and shPLKO cells were treated with or without LPS (100 ng/mL, 12 h) and cultured for 14 days. Colony formation assay was performed for cells. **(M)** The representative images of shSMURF2 and shPLKO patient-derived cells formed tumors in nude mice with or without LPS (10 mg/kg). **(N)** The graph showed the quantified data of tumor weight. **(O)** Immunohistochemistry (IHC) analysis of tumor tissue slides with antibodies against Ki67. Nucleus was stained by hematoxylin. Scale bar, 50 μm **(P)** The proposed model indicates that SMURF2 promotes cell apoptosis through NRF2 inactivation. Data were presented as the mean ± SD from three independent experiments. ∗*p* < 0.05, ∗∗*p* < 0.01, ∗∗∗*p* < 0.001. Scale bar: 10 μm, Scale bar, 50 μm.

We next examined whether SMURF2-mediated degradation of NRF2 contributes to the reduction in protein aggregate formation. IF assays showed that HA-SMURF2 overexpression reduced, while Myc-NRF2 overexpression increased, ub^+^/p62^+^ puncta induced by various stressors (MG132, H_2_O_2_, and LPS). Notably, co-overexpression of HA-SMURF2 and Myc-NRF2 resulted in fewer ub^+^/p62^+^ puncta compared to Myc-NRF2 overexpression alone (Fig. 5A). Similarly, overexpression of Flag-SMURF2 significantly reduced levels of ub and p62. This effect was partially reversed by co-overexpression of Myc-NRF2 (Fig. 5B). Consistently, Flag-SMURF2 overexpression downregulated mRNA levels of NQO1, HO-1, and p62 at the transcriptional level. This reduction was partially mitigated by co-overexpression of Myc-NRF2 (Fig. 5C). Significantly, co-transfection of Flag-SMURF2 with wild-type Myc-NRF2, but not with the NRF2 K555R mutant, significantly reduced levels of ubiquitin and p62 compared to cells transfected with wild-type Myc-NRF2 alone in NRF2-knockdown HEK293T cells (Fig. 5D). Collectively, these findings demonstrate that SMURF2 inhibits protein aggregate formation through K555-specific degradation of NRF2.

We hypothesize that SMURF2 potentially serves as a novel safeguard against constitutive NRF2 activation by mediating NRF2 degradation. Next, flow cytometry analysis revealed that SMURF2 knockdown significantly attenuated intracellular reactive oxygen species (ROS) levels in LN229 cells compared to controls under H_2_O_2_-induced oxidative stress (Fig. 5E, quantified in 5F), while SMURF2 overexpression exacerbated ROS accumulation (Fig. 5F). These results suggest that SMURF2 regulates cellular ROS levels by governing NRF2 stability, thereby influencing cellular antioxidant defenses. To determine whether SMURF2 influences cell survival, we examined apoptosis rates under oxidative stress. SMURF2 knockdown resulted in decreased apoptosis, while HA-SMURF2 overexpression promoted apoptosis (Fig. 5G, quantified in 5H). These findings reveal that SMURF2 upregulation promotes NRF2 degradation, thereby increasing cellular susceptibility to oxidative stress-induced apoptosis. Furthermore, overexpression of Flag-SMURF2 led to increased levels of the pro-apoptotic protein cleaved-caspase-3 under various stressors (MG132, H_2_O_2_, and LPS) (Fig. 5I). Overexpression of Myc-NRF2 reduced cleaved-caspase-3 levels. Interestingly, co-overexpression of Flag-SMURF2 and Myc-NRF2 resulted in higher cleaved-caspase-3 levels than those observed with NRF2 overexpression alone (Fig. 5J). Notably, overexpression of the Myc-NRF2-K555R mutant prevented the upregulation of cleaved-caspase-3 levels (Fig. 5K). We further evaluated the impact of SMURF2 knockdown on cancer cell survival. Colony formation assays revealed that cells expressing shSMURF2 had a higher survival rate than the shPLKO controls under LPS stimulation (Fig. 5L). Next, we used a xenograft BALB/c-nude mouse model to investigate the role of SMURF2 in tumor growth *in vivo*. Patient-derived cells transduced with shPLKO or shSMURF2 were injected into the left and right flanks of each mouse, respectively. Following administration of LPS or PBS at indicated time points, tumor size and weight were significantly increased in the shSMURF2 group compared to the shPLKO group upon LPS treatment (Fig. 5M and N). To assess proliferative activity within these tumors, we performed immunohistochemical staining for Ki67 on harvested tumor tissues. The results demonstrated markedly enhanced proliferation in tumors from the shSMURF2 group, particularly under LPS exposure (Fig. 5O). Collectively, these findings demonstrate that SMURF2 acts as a regulator “brake” on NRF2 mediated pro-survival effects in cancer cells. This newly identified SMURF2- NRF2 regulatory axis provides insights into cellular stress responses and may offer new targets for cancer therapy (Fig. 5P).

### 3.6 SMURF2 promotes NRF2^hi^ patient survival

**Fig. 6:**
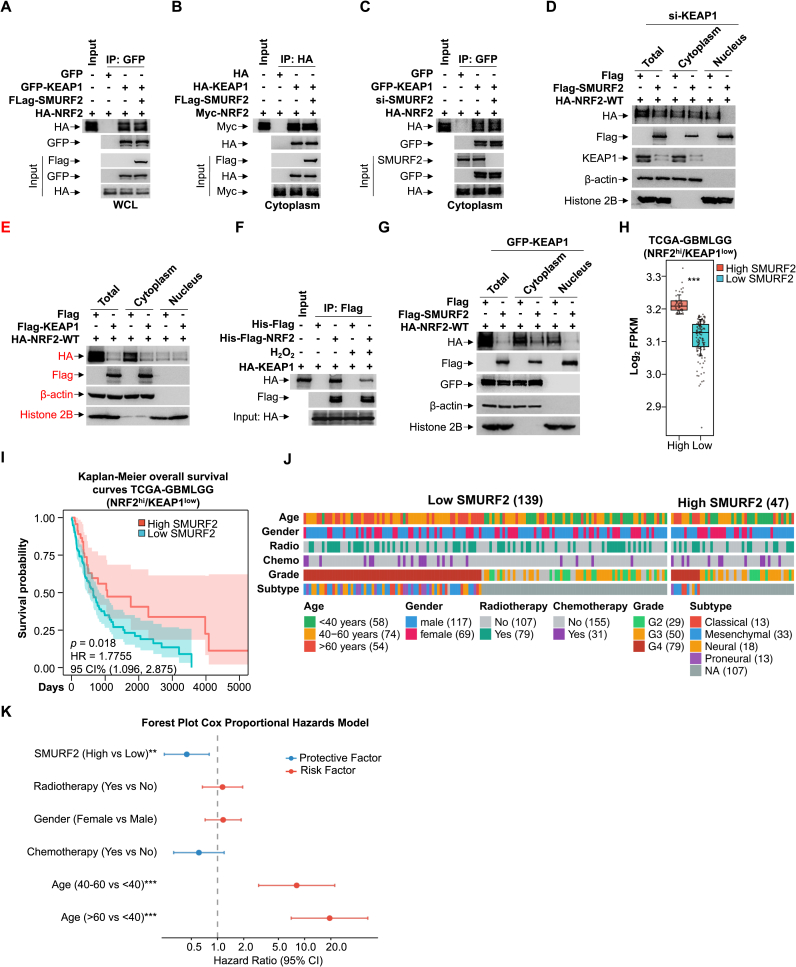
SMURF2 promotes NRF2^hi^ patient survival. **(A)** HA-NRF2 overexpressing HEK293T cells were transfected with GFP or GFP-KEAP1, followed by transfection with Flag or Flag-SMURF2. Co-IP analysis of the interaction between HA-NRF2 and GFP-KEAP1 in WCL. **(B)** Myc-NRF2 overexpressing HEK293T cells were transfected with HA or HA-KEAP1, followed by transfection with Flag or Flag-SMURF2. Co-IP analysis of the interaction between Myc-NRF2 and HA-KEAP1 in the cytoplasm. **(C)** HA-NRF2 overexpressing HEK293T cells were transfected with GFP or GFP-KEAP1, followed by transfection with SMURF2 siRNA or scramble siRNA oligos for 48 h. Co-IP analysis of the interaction between HA-NRF2 and GFP-KEAP1 in the cytoplasm under with or without SMURF2. **(D)** HEK293T cells were initially transfected with KEAP1 siRNA for 48 h, followed by transfection with HA-NRF2-WT, and subsequently transfected with Flag or Flag-SMURF2. Subcellular fractionation was performed to isolate total, nuclear, and cytoplasmic proteins, followed by western blot analysis with indicated antibodies. **(E)** HEK293T cells were first transfected with HA-NRF2-WT. Subsequently, cells were transfected either with Flag or Flag-KEAP1. Subcellular fractionation was performed to isolate total, nuclear, and cytoplasmic proteins, followed by western blot analysis with indicated antibodies. **(F)** GFP-KEAP1 overexpression HEK293T cells were transfected with HA-NRF2-WT, and subsequently transfected with Flag or Flag-SMURF2. Subcellular fractionation was performed to isolate total, nuclear, and cytoplasmic proteins, followed by western blot analysis with indicated antibodies. **(G)** Co-IP assay analysis of the interaction between HA-KEAP1 and His-Flag-NRF2 after treated with or without H_2_O_2_ (200 μM, 2 h) **(H)** Boxplot of SMURF2 expression (log_2_ FPKM) in the NRF2-high/KEAP1-low subset of TCGA-GBMLGG samples. **(I)** Kaplan-Meier survival analysis of TCGA-GBMLGG patients grouped by expression of SMURF2 in the NRF2-high/KEAP1-low subset (*p* = 0.018, HR = 1.7755, log rank test). **(J)** Clinical feature distribution across SMURF2 expression groups. **(K)** Forest Plot of Multivariable Cox Proportional Hazards Analysis in High-Grade Gliomas. Data were presented in three independent experiments. ∗∗*p* < 0.01; ∗∗∗*p* < 0.001.

To define the contribution of SMURF2 relative to the canonical KEAP1 pathway, we first assessed their functional relationship. Co-IP assays showed that modulating SMURF2 levels did not affect the KEAP1-NRF2 interaction in whole-cell or cytoplasmic lysates (Fig. 6A-C), indicating that SMURF2 does not interfere with KEAP1-mediated cytoplasmic sequestration of NRF2. Notably, SMURF2 mediated the degradation of nuclear NRF2 independently of KEAP1, with neither its knockdown nor overexpression affecting this process (Fig. 6D and E). We hypothesized that this independence arises from their distinct subcellular localizations. Subcellular fractionation subsequently confirmed that SMURF2 acts primarily in the nucleus (Fig. 6F). Furthermore, we evaluated pathway dynamics under oxidative stress. H_2_O_2_ treatment significantly disrupted the KEAP1-NRF2 interaction (Fig. 6G), indicating impairment of the primary cytoplasmic axis. In contrast, the SMURF2-mediated degradation of nuclear NRF2 was enhanced under the same conditions (Fig. 3), underscoring its increased functional role when the KEAP1 pathway is compromised.

To evaluate the clinical relevance of SMURF2 as an independent nuclear regulator of NRF2, we analyzed GBM patient data from The Cancer Genome Atlas (TCGA). In the NRF2^hi^/KEAP1^low^ subset (KEAP1 low expression usually leading the constitutive activation of NRF2), patients with high SMURF2 expression exhibited a significantly prolonged overall survival compared to those with low SMURF2 expression (Fig. 6H and I). To determine whether SMURF2 is an independent prognostic factor, we performed multivariable Cox regression analysis, adjusting for age, gender, chemotherapy, and radiotherapy, revealing that SMURF2^hi^ patients exhibited a reduced hazard ratio of mortality, indicating a protective prognostic effect (Fig. 6J and K). This finding suggests that SMURF2 may serve as a favorable prognostic biomarker and potential therapeutic target in NRF2-driven high-grade GBM.

Taken together, our integrated mechanistic and clinical findings establish SMURF2 as a novel, KEAP1-independent, nuclear-negative regulator of NRF2. Its ability to degrade nuclear NRF2 and its association with improved survival specifically in tumors with compromised KEAP1 function highlight SMURF2 not only as a compelling prognostic indicator but also as a context-specific therapeutic target for NRF2-constitutive activation cancers.

## 4 Discussion and conclusion

**Fig. 7:**
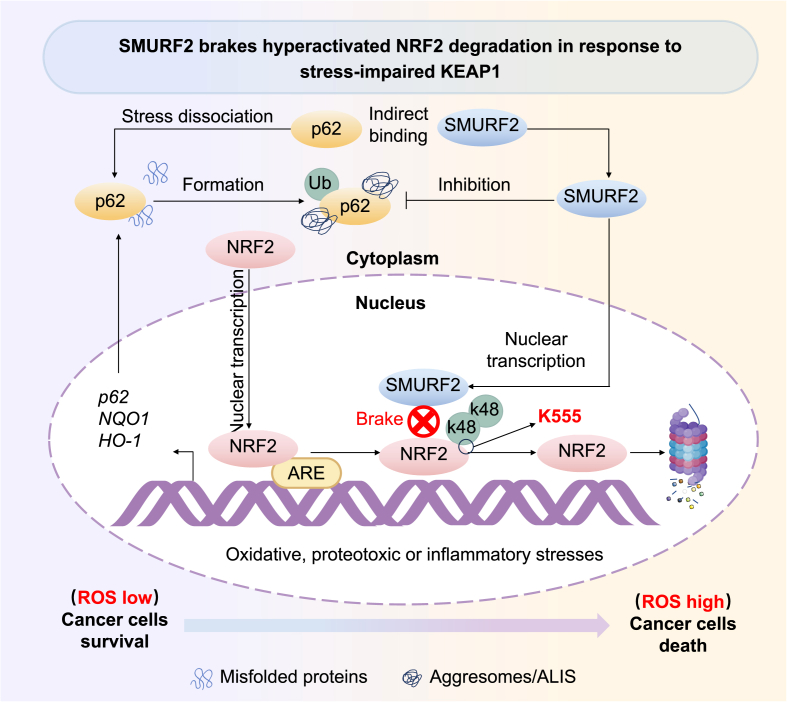
**SMURF2 mediates the degradation of NRF2 during stress response management.** The revised schematic model illustrates that under stress conditions, SMURF2 translocate to the nucleus independent of KEAP1 function and acts as a regulatory brake by ubiquitinating hyperactivated NRF2 for degradation, thereby attenuating antioxidant effects in tumor cells and promoting GBM cell apoptosis.

Constitutive activation of NRF2 significantly contributes to tumor progression in neoplasms and severely compromises the efficacy of chemo- and radiotherapy. Our study uncovered a novel role of SMURF2 as a nuclear “brake” of NRF2, which regulates anti-oxidative and anti-proteotoxic stress responses. As a tumor suppressor, SMURF2 specifically degrades nuclear NRF2 (independent of the canonical KEAP1 pathway, which facilitates the degradation of cytoplasmic NRF2) to inhibit its mediated cytoprotective responses in tumor cells. Additionally, multivariable Cox regression analysis confirmed SMURF2 as an independent favorable prognostic factor (Fig. 6J and K), suggesting a direct association between SMURF2-mediated tumor suppression in highly expressed NRF2 patients. Notably, we observed that under conditions where stress impairs KEAP1 function, SMURF2 acts as a brake by ubiquitinating and degrading hyperactivated NRF2, thereby inhibiting NRF2-driven sequestration of cytotoxic misfolded aggresomes—a process critical for disrupting a key protective mechanism in cancer cells (Fig. 7). Thus, our findings highlight the tumor-suppressive role of SMURF2 in GBM adaptive NRF2 pathways by mediating proteotoxic stress.

Previous studies have documented SMURF2's diverse roles in cancer, including interactions with KAP1, YY1, and HIF-1α [17,39,40]. To evaluate whether these established targets contribute to SMURF2-mediated stress sensitivity in our model, we examined their interactions with SMURF2 under basal and stress conditions. Consistent with previous reports, SMURF2 interacted with KAP1, YY1, and HIF1α under both basal and stress conditions. However, these associations were not enhanced by stress (data not shown), suggesting that they may not be the primary mediators of SMURF2's stress-responsive functions in our system. Notably, in this study, the SMURF2-NRF2 interaction was significantly strengthened under oxidative stress conditions, as observed for both endogenous proteins and exogenously overexpressed constructs, demonstrating its stimulus-specific recruitment (Fig. 2B and C). Ubiquitination-mediated degradation of transcription factors is recognized as an efficient way to regulate transcription and terminate cellular signaling [[41], [42], [43]]. Under stress conditions, SMURF2 translocate to the nucleus where it ubiquitinates NRF2 (Fig. 4). This nuclear-specific mechanism of regulation exemplifies the critical role of spatial control in cellular processes. A similar spatial control mechanism is observed in the TRIM26-mediated degradation of IRF3 in the nucleus, which prevents excessive antiviral immune responses [38]. Importantly, NLS-mutated SMURF2 (incapable of nuclear entry) fails to degrade NRF2, definitively proving that nuclear localization is essential for this function. Interestingly, previous research has indicated the role of 14-3-3 proteins in restricting SMURF2's cytoplasmic localization [44]. We hypothesize that the accumulation of SMURF2 in the cytoplasm at a basal level (as shown in Fig. 3) may involve sequestration mediated by these 14-3-3 proteins, which could potentially release NRF2 from nuclear suppression under normal conditions. However, under oxidative stress, SMURF2 overcomes this cytoplasmic retention and translocate to the nucleus, where it can access and degrade nuclear NRF2. Further investigations revealed that SMURF2 specifically targets NRF2 for ubiquitination at lysine 555 in the nucleus (Fig. 3, Fig. 4). This specificity underscores the precise nature of SMURF2's regulatory function on NRF2 under stress conditions. Thus, the SMURF2-NRF2 axis represents the predominant pathway regulated by stress in our system, driving the suppression of cytoprotective responses and consequent sensitization of GBM cells to stress-induced death.

Multiple pathways regulate NRF2 stability, two well-characterized cytoplasmic systems predominate: the KEAP1-CUL3 axis maintaining basal degradation, and the GSK-3β/β-TrCP-CUL1 axis terminating stress responses through nuclear export [7,[45], [46], [47]]. Our study identifies that under oxidative/protein toxicity stress, SMURF2 forms another regulatory pathway via nuclear translocation. Here, we uncover a spatially distinct third mechanism: stress-induced SMURF2 nuclear translocation enables direct ubiquitination and degradation of nuclear NRF2. Notably, SMURF2 acts as a late-phase nuclear checkpoint that constrains pathological NRF2 overactivation (data not shown). This compartment-specific degradation positions SMURF2 as a stress-inducible nuclear termination switch that depletes nuclear NRF2 pools (Fig. 2, Fig. 3, Fig. 4), suppressing cytoprotective gene transcription to override adaptive survival signals. By executing delayed degradation when cytoplasmic pathways are saturated, this mechanism resolves the conundrum of persistent nuclear NRF2 in cancers. The functional consequences of disrupting this nuclear brake are severe. The loss of SMURF2 results in the abnormal accumulation of nuclear NRF2, which, by continuously activating p62 (a known mediator of aggregate assembly) [22], promotes protein aggregate formation and enhances chemoresistance (Fig. 3, Fig. 5). Concurrently, excessive nuclear NRF2 stabilization upregulates antioxidant genes such as NQO1 and HO-1, reinforcing the tumor cell's antioxidant capacity. Conversely, SMURF2 overexpression exerts dual cytotoxic effects through nuclear NRF2 degradation: it disrupts the misfolded protein sequestration-clearance mechanism (by reducing p62 activation) and suppresses the antioxidant defense system, ultimately leading to increased intracellular ROS levels and inducing apoptosis (Fig. 1, Fig. 5).

Our findings establish SMURF2 as a promising therapeutic target for NRF2-driven cancers; however, its clinical translation requires consideration of its multifaceted roles. A primary concern is SMURF2's established function in the TGF-β pathway, where it regulates receptors and SMAD proteins [48]. Nevertheless, key features of the SMURF2-NRF2 axis revealed here suggest a viable path toward selective targeting. First, the spatial compartmentalization of SMURF2's functions provides a fundamental rationale: SMURF2 regulates TGF-β components predominantly in the cytoplasm [48], whereas its degradation of NRF2 is a stress-induced event confined to the nucleus (Fig. 3). Second, this nuclear interaction is dynamically enhanced under the oxidative/proteotoxic stress characteristic of tumors, indicating context-dependent activation. Third, our prognostic analysis indicates that the benefit of high SMURF2 is most pronounced in NRF2-high patients, supporting biomarker-guided stratification to maximize efficacy and minimize off-target exposure. Therefore, future therapeutic strategies could focus on precisely augmenting the nuclear SMURF2-NRF2 axis by promoting SMURF2 nuclear activity or mimicking its nuclear function, while sparing its cytoplasmic roles. Comprehensive preclinical evaluation of effects on TGF-β-dependent processes remains essential. Ultimately, leveraging this spatial and contextual specificity may enable the development of selective therapies against NRF2-addicted cancers.

In conclusion, SMURF2 facilitates the degradation of NRF2 through ubiquitination in the nucleus, thereby preventing excessive antioxidant responses induced by constitutive activation of NRF2(which could lead to tumor proliferation or drug resistance), and promoting tumor cell apoptosis. These findings lay a crucial theoretical foundation and practical direction for developing novel cancer treatment strategies targeting constitutive NRF2 activation.

## CRediT authorship contribution statement

**Wanting Xu:** Conceptualization, Data curation, Formal analysis, Writing – original draft, Writing – review & editing. **Lei Dong:** Formal analysis, Funding acquisition, Supervision, Writing – review & editing. **Jiaqian Li:** Formal analysis, Writing – review & editing. **Shuai Fan:** Formal analysis, Funding acquisition, Writing – review & editing. **Yadong Wang:** Writing – review & editing. **Qin Xia:** Conceptualization, Data curation, Formal analysis, Funding acquisition, Investigation, Supervision.

## Declaration of competing interest

We have read and understood your journal's policies, and we believe that neither the manuscript nor the study violates any of these. There are no conflicts of interest to declare.

## Data Availability

I have shared the link to my data at the Attach File step.

## References

[1] Torrente L., DeNicola G.M. (2022). Targeting NRF2 and its downstream processes: opportunities and challenges. Annu. Rev. Pharmacol. Toxicol..

[2] Jena K.K. (2018). TRIM16 controls assembly and degradation of protein aggregates by modulating the p62-NRF2 axis and autophagy. Embo j.

[3] McMahon M., Thomas N., Itoh K., Yamamoto M., Hayes J.D. (2004). Redox-regulated turnover of Nrf2 is determined by at least two separate protein domains, the redox-sensitive Neh2 degron and the redox-insensitive Neh6 degron. J. Biol. Chem..

[4] Adinolfi S. (2023). The KEAP1-NRF2 pathway: targets for therapy and role in cancer. Redox Biol..

[5] Tong K.I. (2007). Different electrostatic potentials define ETGE and DLG motifs as Hinge and latch in oxidative stress response. Mol. Cell Biol..

[6] Cuadrado A. (2015). Structural and functional characterization of Nrf2 degradation by glycogen synthase kinase 3/β-TrCP. Free Radic. Biol. Med..

[7] Zhang J., Zhang M., Tatar M., Gong R. (2025). Keap1-independent Nrf2 regulation: a novel therapeutic target for treating kidney disease. Redox Biol..

[8] Padmanabhan B. (2006). Structural basis for defects of Keap1 activity provoked by its point mutations in lung cancer. Mol. Cell.

[9] Pölönen P. (2019). Nrf2 and SQSTM1/p62 jointly contribute to mesenchymal transition and invasion in glioblastoma. Oncogene.

[10] Hellyer J.A., Padda S.K., Diehn M., Wakelee H.A. (2021). Clinical implications of KEAP1-NFE2L2 mutations in NSCLC. J. Thorac. Oncol..

[11] Sanchez-Vega F. (2018). Oncogenic signaling pathways in the cancer genome atlas. Cell.

[12] Chowdhry S. (2013). Nrf2 is controlled by two distinct β-TrCP recognition motifs in its Neh6 domain, one of which can be modulated by GSK-3 activity. Oncogene.

[13] Liang J. (2015). Expression of β-transducin repeat-containing E3 ubiquitin protein ligase in human glioma and its correlation with prognosis. Oncol. Lett..

[14] Fu L., Cui C.P., Zhang X., Zhang L. (2020). The functions and regulation of smurfs in cancers. Semin. Cancer Biol..

[15] Blank M. (2012). A tumor suppressor function of Smurf2 associated with controlling chromatin landscape and genome stability through RNF20. Nat. Med..

[16] Ramkumar C. (2012). Smurf2 regulates the senescence response and suppresses tumorigenesis in mice. Cancer Res..

[17] Ramkumar C. (2013). Smurf2 suppresses B-cell proliferation and lymphomagenesis by mediating ubiquitination and degradation of YY1. Nat. Commun..

[18] Du J.X. (2011). The E3 ubiquitin ligase SMAD ubiquitination regulatory factor 2 negatively regulates Krüppel-like factor 5 protein. J. Biol. Chem..

[19] Dong J.T., Chen C. (2009). Essential role of KLF5 transcription factor in cell proliferation and differentiation and its implications for human diseases. Cell. Mol. Life Sci..

[20] Park J. (2017). Misfolded polypeptides are selectively recognized and transported toward aggresomes by a CED complex. Nat. Commun..

[21] Fujita K., Maeda D., Xiao Q., Srinivasula S.M. (2011). Nrf2-mediated induction of p62 controls toll-like receptor-4-driven aggresome-like induced structure formation and autophagic degradation. Proc. Natl. Acad. Sci. USA.

[22] Vargas J.N.S., Hamasaki M., Kawabata T., Youle R.J., Yoshimori T. (2023). The mechanisms and roles of selective autophagy in mammals. Nat. Rev. Mol. Cell Biol..

[23] Tabrizi S.J. (2022). Potential disease-modifying therapies for huntington's disease: lessons learned and future opportunities. Lancet Neurol..

[24] Guo Y. (2024). ALS-linked SOD1 mutations impair mitochondrial-derived vesicle formation and accelerate aging. Redox Biol..

[25] Dayton R.D. (2013). Selective forelimb impairment in rats expressing a pathological TDP-43 25 kDa C-terminal fragment to mimic amyotrophic lateral sclerosis. Mol. Ther..

[26] Gal J. (2009). Sequestosome 1/p62 links familial ALS mutant SOD1 to LC3 via an ubiquitin-independent mechanism. J. Neurochem..

[27] Bersuker K., Brandeis M., Kopito R.R. (2016). Protein misfolding specifies recruitment to cytoplasmic inclusion bodies. J. Cell Biol..

[28] Zhang Y.J. (2009). Aberrant cleavage of TDP-43 enhances aggregation and cellular toxicity. Proc. Natl. Acad. Sci. USA.

[29] Riley B.E., Kaiser S.E., Kopito R.R. (2011). Autophagy inhibition engages Nrf2-p62 Ub-associated signaling. Autophagy.

[30] Komatsu M. (2010). The selective autophagy substrate p62 activates the stress responsive transcription factor Nrf2 through inactivation of Keap1. Nat. Cell Biol..

[31] Wiesner S. (2007). Autoinhibition of the HECT-type ubiquitin ligase Smurf2 through its C2 domain. Cell.

[32] Zhang W. (2023). SMURF2 predisposes cancer cell toward ferroptosis in GPX4-independent manners by promoting GSTP1 degradation. Mol. Cell.

[33] Pohl C., Dikic I. (2019). Cellular quality control by the ubiquitin-proteasome system and autophagy. Science.

[34] Xiong Y. (2022). ADAP restraint of STAT1 signaling regulates macrophage phagocytosis in immune thrombocytopenia. Cell. Mol. Immunol..

[35] Zhang D.D. (2006). Mechanistic studies of the Nrf2-Keap1 signaling pathway. Drug Metab. Rev..

[36] Sun Z., Zhang S., Chan J.Y., Zhang D.D. (2007). Keap1 controls postinduction repression of the Nrf2-mediated antioxidant response by escorting nuclear export of Nrf2. Mol. Cell Biol..

[37] Wu T. (2014). Hrd1 suppresses Nrf2-mediated cellular protection during liver cirrhosis. Genes Dev..

[38] Wang P., Zhao W., Zhao K., Zhang L., Gao C. (2015). TRIM26 negatively regulates interferon-β production and antiviral response through polyubiquitination and degradation of nuclear IRF3. PLoS Pathog..

[39] Addison J.B. (2015). KAP1 promotes proliferation and metastatic progression of breast cancer cells. Cancer Res..

[40] Youssef E., Zhao S., Purcell C., Olson G.L., El-Deiry W.S. (2024). Targeting the SMURF2-HIF1α axis: a new frontier in cancer therapy. Front. Oncol..

[41] Hodáková Z. (2023). Cryo-EM structure of the chain-elongating E3 ubiquitin ligase UBR5. Embo j.

[42] Deng M., Hochstrasser M. (2006). Spatially regulated ubiquitin ligation by an ER/nuclear membrane ligase. Nature.

[43] Khmelinskii A. (2014). Protein quality control at the inner nuclear membrane. Nature.

[44] Emanuelli A. (2019). Altered expression and localization of tumor suppressive E3 ubiquitin ligase SMURF2 in human prostate and breast cancer. Cancers (Basel).

[45] Huang W. (2024). Inhibition of MST1 ameliorates neuronal apoptosis via GSK3β/β-TrCP/NRF2 pathway in spinal cord injury accompanied by diabetes. Redox Biol..

[46] Huang S. (2022). Hepatic TGFβr1 deficiency attenuates Lipopolysaccharide/D-Galactosamine-Induced acute liver failure through inhibiting GSK3β-Nrf2-Mediated hepatocyte apoptosis and ferroptosis. Cell. Mol. Gastroenterol. Hepatol..

[47] Patibandla C. (2024). Inhibition of glycogen synthase kinase-3 enhances NRF2 protein stability, nuclear localisation and target gene transcription in pancreatic beta cells. Redox Biol..

[48] Kavsak P. (2000). Smad7 binds to Smurf2 to form an E3 ubiquitin ligase that targets the TGF beta receptor for degradation. Mol. Cell.

