# Supplementary material for: SMURF2 attenuates NRF2-driven tumor progression by acting as a nuclear brake on NRF2 during cellular stress
Source: Redox Biol. 2026 Feb 28;92:104102. doi: 10.1016/j.redox.2026.104102 (PMC13084320; doi:10.1016/j.redox.2026.104102)

**Supplementary Figure 1. SMURF2 prohibits the stress-mediated formation of ub⁺/p62⁺ aggresomes**

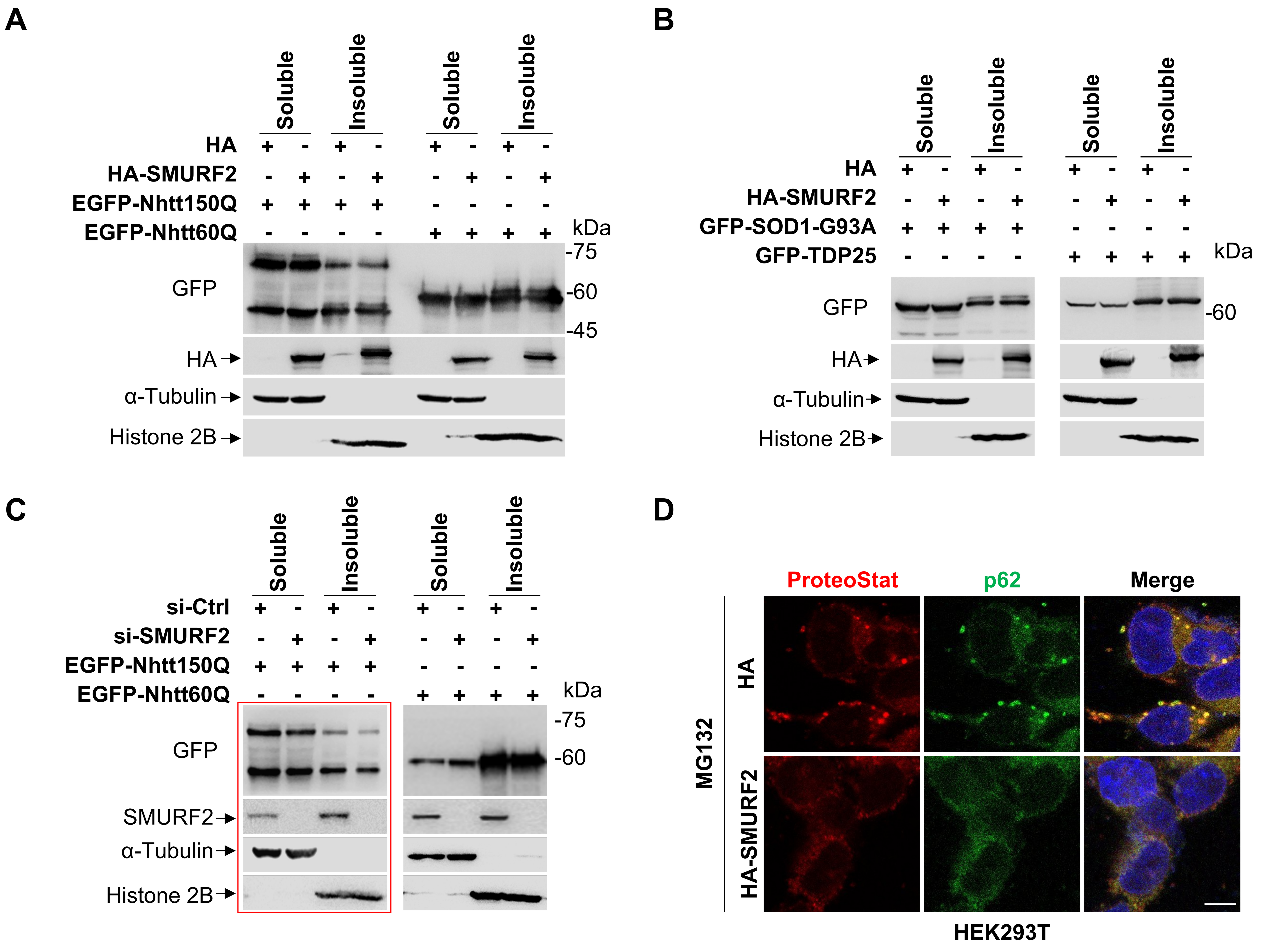

**Supplementary Figure 2. p62 indirectly interacts with SMURF2**

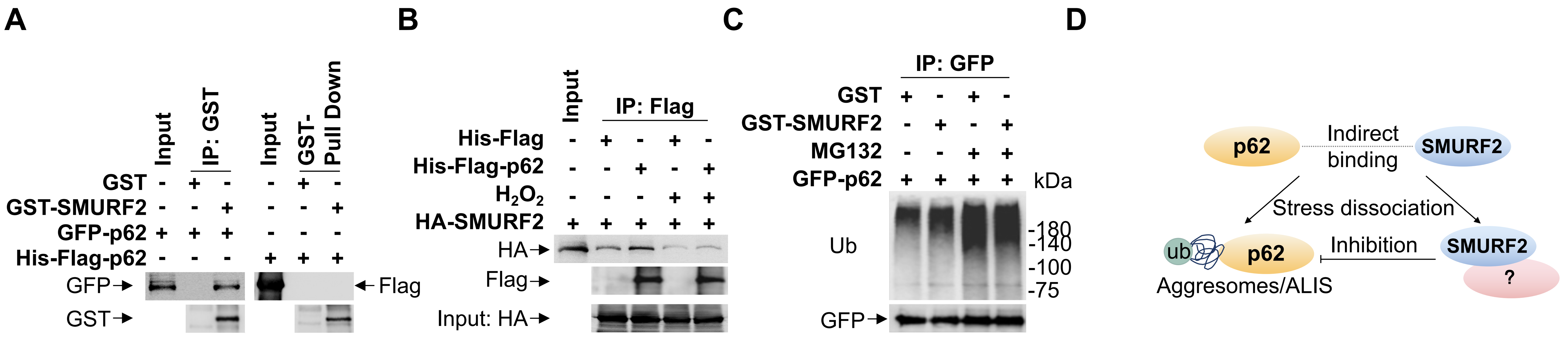

**Supplementary Figure 3. SMURF2 facilitates NRF2 proteasomal degradation in response to cellular stress**

**A**

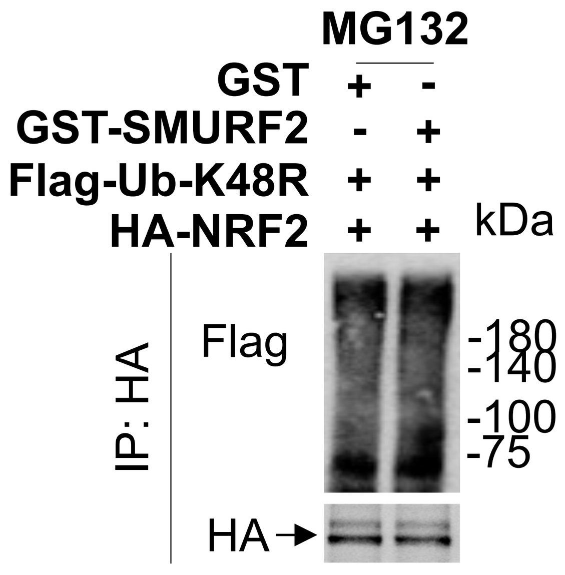


**B**

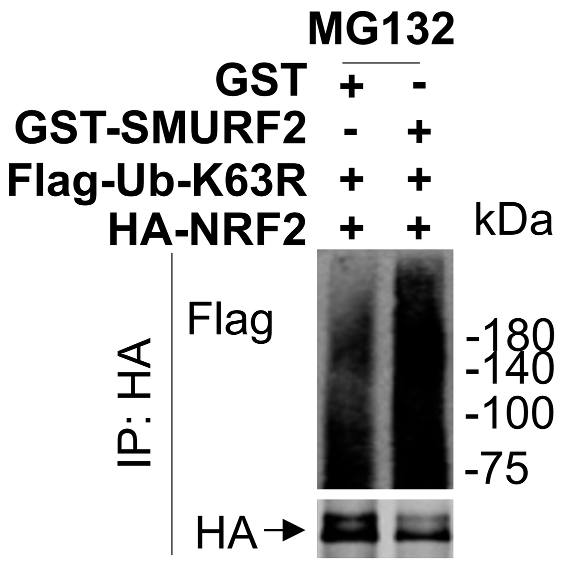

**Supplementary Figure 4. SMURF2 nuclear translocation facilitates the degradation of NRF2 within the nucleus**

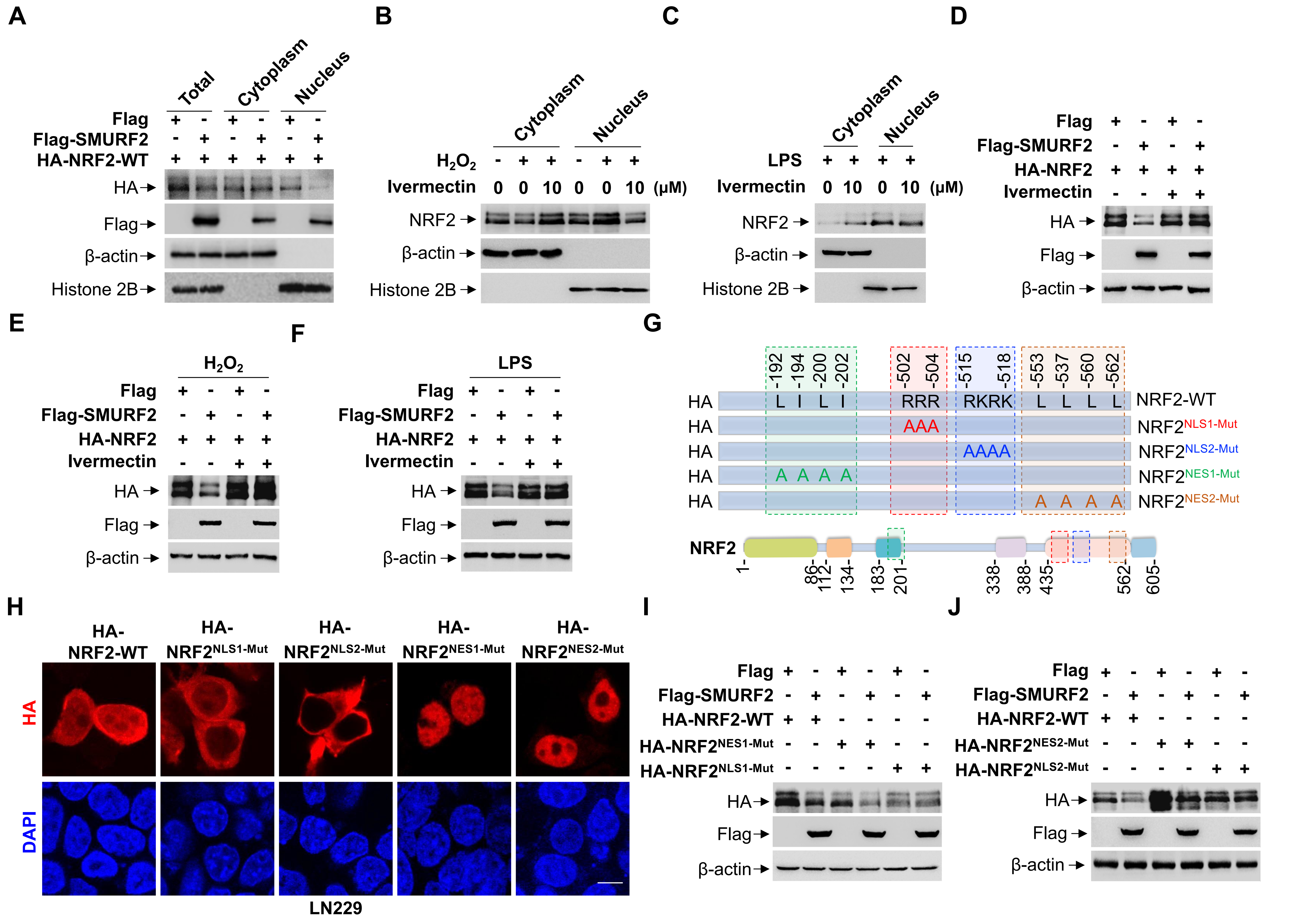

Supplement: Multimedia component 1 [file mmc1.pdf]
