# Supplementary material for: SMURF2 attenuates NRF2-driven tumor progression by acting as a nuclear brake on NRF2 during cellular stress
Source: Redox Biol. 2026 Feb 28;92:104102. doi: 10.1016/j.redox.2026.104102 (PMC13084320; doi:10.1016/j.redox.2026.104102)

Figure 1. SMURF2 prohibits the stress-mediated formation of ub+/p62+ aggresomes

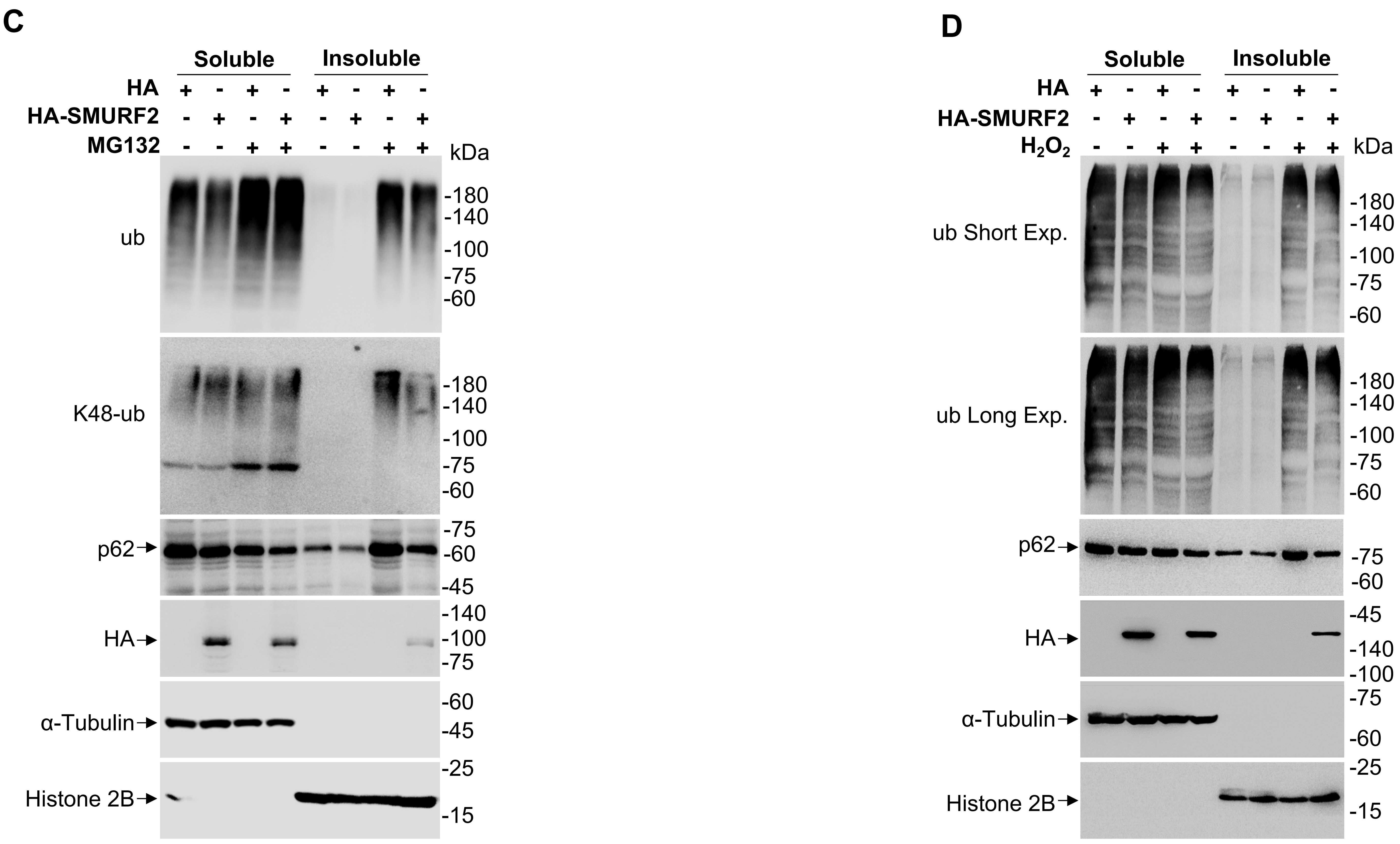

Figure 2. SMURF2 facilitates NRF2 proteasomal degradation in response to cellular stress

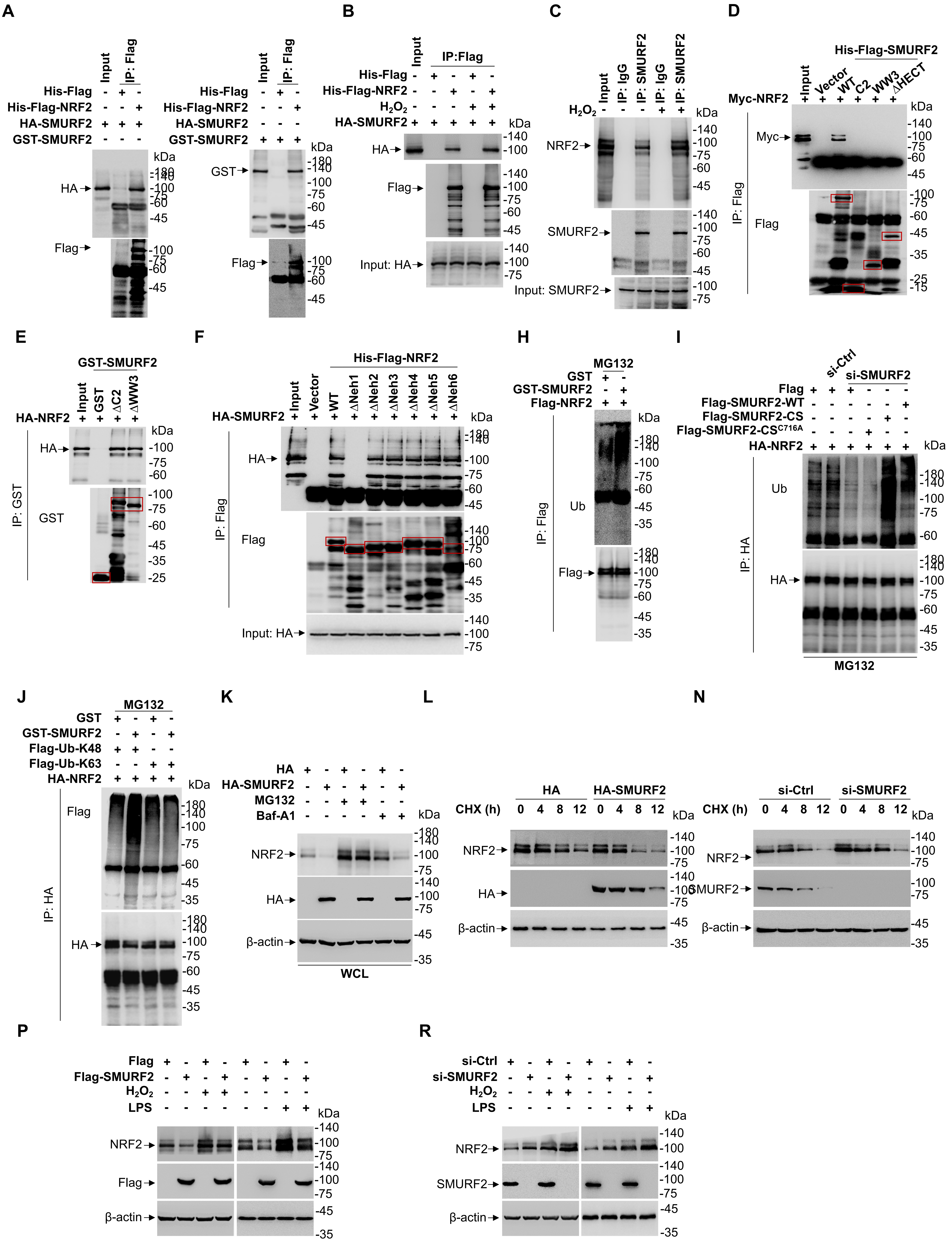

Figure 3. SMURF2 nuclear translocation facilitates the degradation of NRF2 within the nucleus

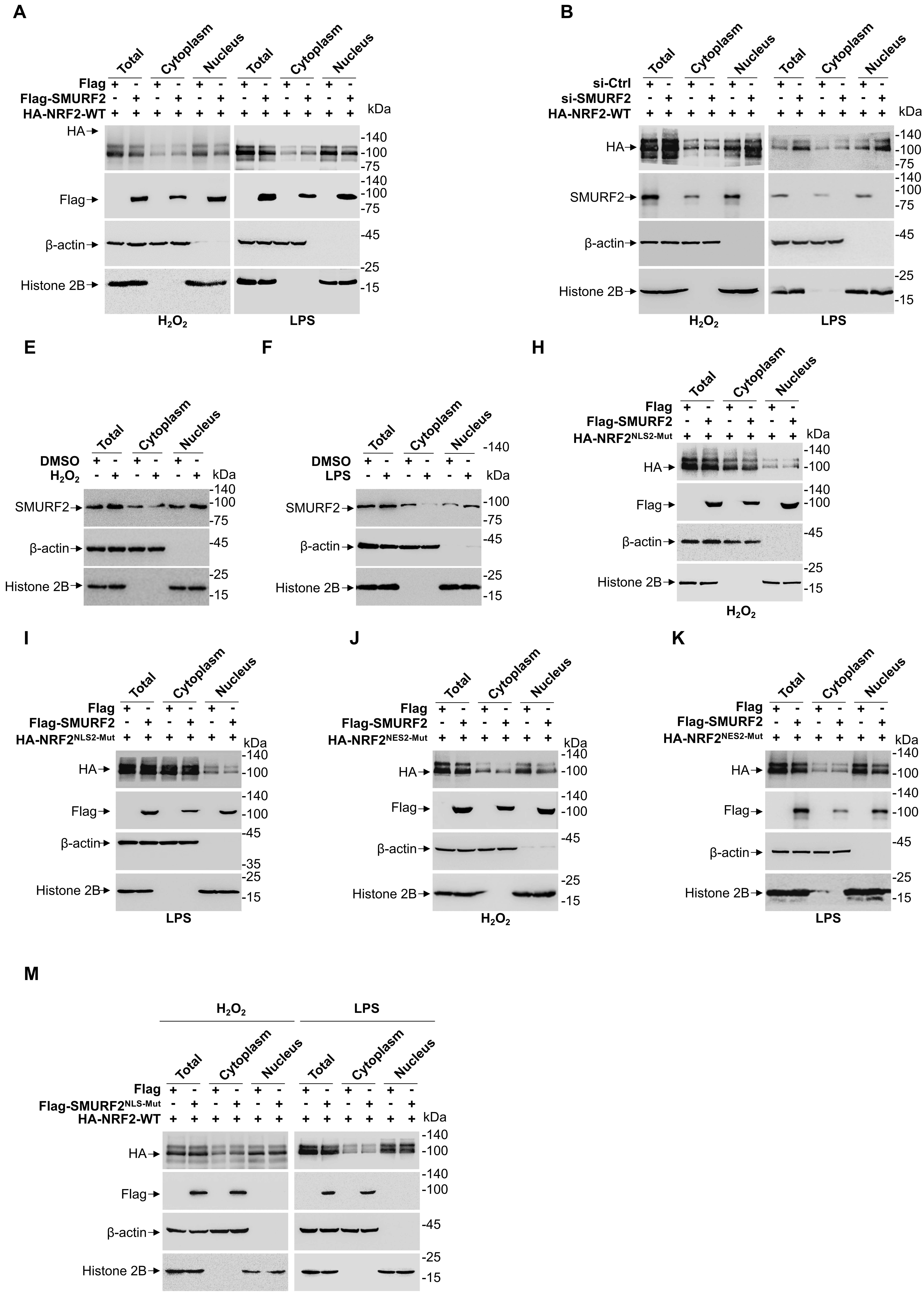

Figure 4. SMURF2 ubiquitinates NRF2 at K555 in the nucleus for its degradation

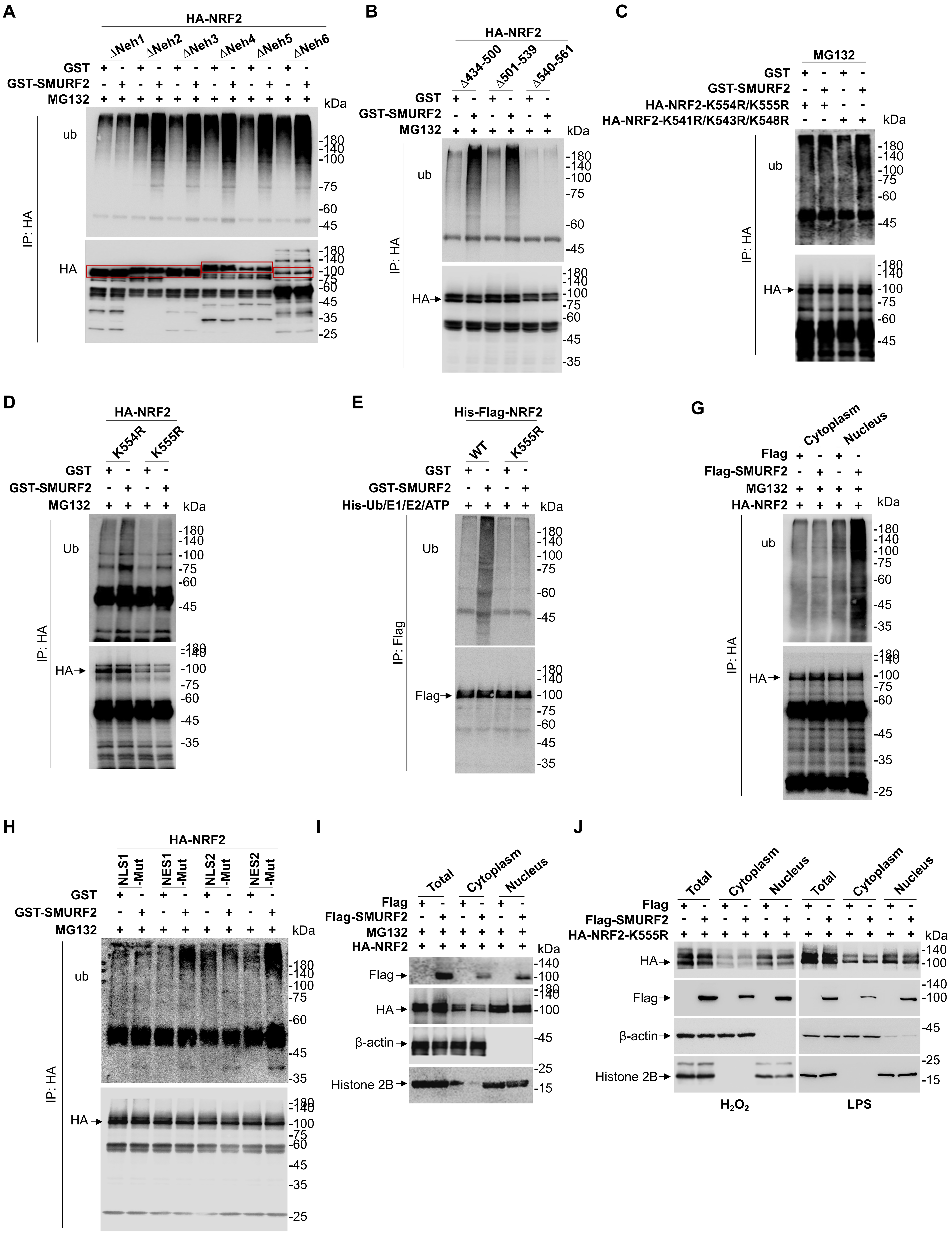

Figure 5. SMURF2 promotes cell apoptosis through NRF2 inactivation

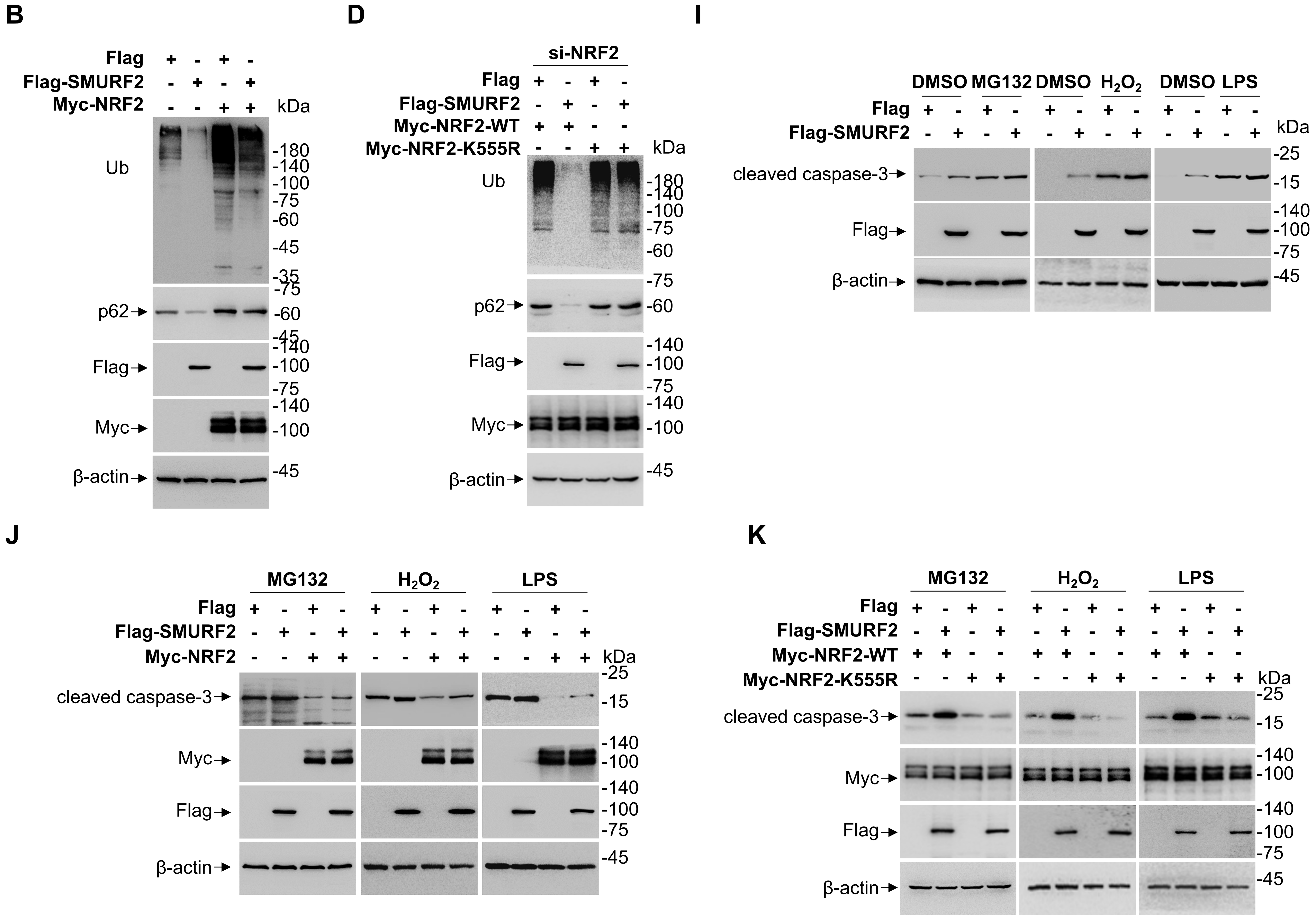

Figure 6. SMURF2 promotes NRF2^{hi} patient survival

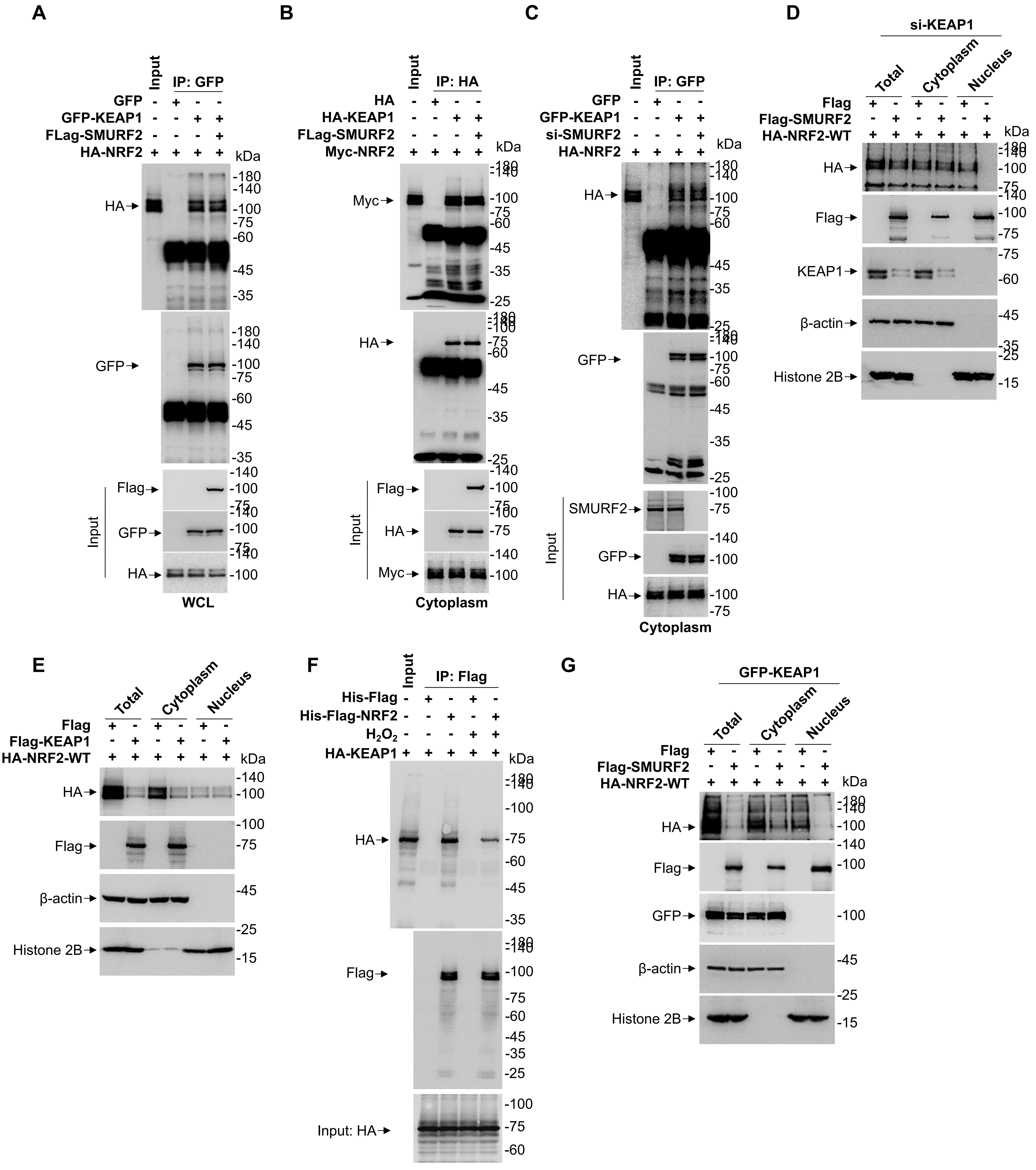

Supplement: Multimedia component 2 [file mmc2.pdf]
